# WNT4 overexpression and secretion in thymic epithelial tumors drive an autocrine loop in tumor cells *in vitro*

**DOI:** 10.3389/fonc.2022.920871

**Published:** 2022-07-29

**Authors:** Xiaonan Zhang, Berthold Schalke, Krisztian Kvell, Katharina Kriegsmann, Mark Kriegsmann, Thomas Graeter, Gerhard Preissler, German Ott, Katrin Kurz, Elena Bulut, Philipp Ströbel, Alexander Marx, Djeda Belharazem

**Affiliations:** ^1^ Institute of Pathology and Medical Research Center, University Medical Centre Mannheim, University of Heidelberg, Mannheim, Germany; ^2^ Department of Neurology, University of Regensburg, Regensburg, Germany; ^3^ Department of Pharmaceutical Biotechnology, Faculty of Pharmacy, University of Pecs, Pecs, Hungary; ^4^ Department of Hematology, Oncology and Rheumatology, University Hospital Heidelberg, Heidelberg, Germany; ^5^ Translational Lung Research Centre Heidelberg, German Centre for Lung Research, Heidelberg, Germany; ^6^ Institute of Pathology, University Hospital Heidelberg, Heidelberg University, Heidelberg, Germany; ^7^ Department of Thoracic Surgery, University Medical Centre Erlangen, Erlangen, Germany; ^8^ Department of Thoraxic Surgery, Clinic Schillerhöhe, Robert-Bosch-Hospital, Gerlingen, Löwenstein, Germany; ^9^ Department of Clinical Pathology, Robert-Bosch-Hospital, Stuttgart, Germany; ^10^ Dr. Margarete Fischer-Bosch Institute for Clinical Pharmacology, Robert-Bosch-Hospital, Stuttgart, Germany; ^11^ Department of Thoraxic Surgery, Thoraxklinik at Heidelberg University Hospital, Heidelberg, Germany; ^12^ Institute of Pathology, University Medical Center Göttingen, University of Göttingen, Göttingen, Germany

**Keywords:** thymoma, thymic carcinoma, primary epithelial cells, WNT4/FZD6, JNK, NF-κB and AKT noncanonical pathway

## Abstract

**Background:**

WNT4-driven non-canonical signaling is crucial for homeostasis and age-related involution of the thymus. Abnormal WNT signaling is important in many cancers, but the role of WNT signaling in thymic tumors is largely unknown.

**Materials & Methods:**

Expression and function of WNT4 and FZD6 were analyzed using qRT–PCR, Western blot, ELISA, in biopsies of non-neoplastic thymi (NT), thymoma and thymic carcinomas. ShRNA techniques and functional assays were used in primary thymic epithelial cells (pTECs) and TC cell line 1889c. Cells were conventionally (2D) grown and in three-dimensional (3D) spheroids.

**Results:**

In biopsy, WHO classified B3 thymomas and TCs showed increased WNT4 expression compared with NTs. During short-term 2D culture, WNT4 expression and secretion declined in neoplastic pTECs but not in 3D spheroids or medium supplemented with recombinant WNT4 cultures. Under the latter condition, the growth of pTECs was accompanied by increased expression of non-canonical targets RAC1 and JNK. Down-regulation of WNT4 by shRNA induced cell death in pTECs derived from B3 thymomas and led to decreased RAC1, but not JNK protein phosphorylation. Pharmacological inhibition of NF-κB decreased both RAC1 and JNK phosphorylation in neoplastic pTECs.

**Conclusions:**

Lack of the age-related decline of non-canonical WNT4 expression in TETs and restoration of declining WNT4 expression through exogeneous WNT4 or 3D culture of pTECs hints at an oncogenic role of WNT4 in TETs and is compatible with the WNT4 autocrine loop model. Crosstalk between WNT4 and NF-κB signaling may present a promising target for combined interventions in TETs.

## Introduction

Thymomas and thymic carcinomas (TCs) are organo-specific thymic epithelial tumors (TETs) (2). Thymomas primarily comprise histological types A, AB, B1, B2, and B3 according to the WHO classification (3). They mostly maintain thymic functions and commonly exhibit autoimmune features (4). Thymic carcinomas belong to aggressive thymic tumors and resemble histological carcinomas originating in non-thymic organs (5). The group of TCs includes several new sub-types: micronodular TC, hyalinizing clear cell carcinoma, and thymic sebaceous carcinoma, which are grouped with other rare TCs (6). Thymic squamous cell carcinoma (TSQCC) (7), the most common TC subtype, has an inferior prognosis than thymomas (8) and lacks thymus-specific functions. Genetic alterations of thymomas and TSQCCs are significantly different from squamous cell carcinomas of the head, neck, and lung (9). The molecular pathology of TETs has only partially been resolved (10). Previous studies have demonstrated that maintenance and functional integrity of the stroma in the normal thymus requires stimulation *via* Notch, bone morphogenetic protein (BMP), and WNT signaling pathways (11–13).

WNT signaling controls multiple biological processes, including proliferation, fate specification, polarity, migration, and stemness, and has also been associated with various human cancers (14, 15). WNT proteins are a family of 19 glycoproteins that can either be tethered to the plasma membrane or exit the cell *via* multiple routes (16). WNT signaling is divided into β-catenin-dependent (canonical) and β-catenin-independent (non-canonical) pathways, which are further divided into i) Planar Cell Polarity (PCP) pathway (activating DSH and RAC1, which in turn activates JUN kinase (JNK) (17)), and ii) WNT/Ca^2+^ pathway based on DSH, DAAM1 and RHO activation, which in turn activates Rho kinase (ROCK) (18–20).

WNT signaling plays a key role in the development of the thymus, and expression levels of WNT ligands (particularly WNT4) decrease during thymic involution in mice and humans (19, 21–23). WNT signaling also regulates T-cell development in the thymus (24). The secretion of WNT ligands mainly depends on acylation by Porcupine (PORCN) (18). WNT4 is secreted from normal thymic epithelial cells and activates a signaling network *via* G-protein-dependent Frizzled receptors in an autocrine manner (25). In mouse models, premature thymic involution is induced upon downregulation of WNT signaling (26). Therefore, the maintenance of the homeostasis of thymic epithelial cells requires WNT signaling (27) and decreased expression of WNT proteins or increased levels of WNT inhibitors is associated with TEC senescence (23). Nevertheless, the signaling mechanisms that regulate thymic involution are incompletely understood (25). Among the known requirements that help maintain the functional integrity of the normal thymic stroma are Notch, BMP, and WNT signaling, and there is evidence that decreasing WNT signaling, including WNT4 signaling, could contribute to age-related thymic involution in humans (11–13, 28, 29). By contrast, whether WNT pathways, specifically WNT4 signaling, play a role in human TETs has not been elucidated. Further motivation to study WNT4 in TETs resulted from the observation in the TCGA study (10) that single nucleotide aberrations strongly hint at the operation of aging-related oncogenic mechanisms in TETs. Finally, since WNT signaling interacts dynamically with the tumor micro-environment (30), we extended our study of WNT4 to 3D TET spheroids that were enriched with or without an extracellular matrix.

## Materials and methods

### Patients

The clinical characteristics of the 82 patients with thymomas, thymic carcinomas, and 21 normal thymi are summarized in **Table 1**. The study was approved by the local Ethics Committee (approval #2009-290N-MA/2010 and 2018-516N-MA).

**Table 1:** Characteristics of the thymoma and thymic carcinoma patients and non-neoplastic adult and pediatric control from cardiac surgery patients studied for WNT ligands, frizzled receptors and WNT inhibitors.

Diagnosis	N	Age range (y)	Sex(m:f)	Stage(I–IV)	MG+ (%)
Type A	9	36–81	4:5	I (n = 4) II (n = 5)	0
Type AB	25	26–77	15:10	I (n = 18) II (n = 7)	9 (40.9%)
Type B2	18	21–81	10:8	I (n = 3) II (n = 6) III (n = 5) IV (n = 4)	8 (44.4%)
Type B3	19	41–76	8:11	I (n = 5) II (n = 4) III (n = 8) IV (n = 2)	4 (21.05%)
TSQCC	11	32–74	6:5	I (n = 5) II (n = 3) III (n = 1) IV (n = 2)	0
CT	6	0–10	4:2	–	–
NT	15	28–82	6:9	–	–

Thymoma type A, AB, B2, and B3 (3); TSQCC: thymic squamous cell carcinoma; MG+ (%): percentage of patients with myasthenia gravis; stage, according to Masaoka-Koga (31); CT, (normal) childhood thymuses; NT, normal (adult) thymuses.

### Primary thymic epithelial cells (pTECs) and cell lines

pTECs were prepared from 5 type AB and 3 type B3 thymomas (henceforth called AB-pTECs and B3-pTECs, respectively) and cultured as described (4, 9). Shortly, cell suspensions were prepared by several rounds of liberase II digestion of tissue fragments and grown at 37°C on uncoated 10 cm tissue plastic dishes (Becton & Dickinson, Heidelberg, Germany) in RPMI 1640 with 2 g/L glucose, 25 mM HEPES, 200 mM L-Glutamine, 50 U/ml penicillin, 50 µg/ml streptomycin, and 10% calf serum (Sigma Aldrich, Germany). The medium was changed every 4 days. The epithelial cell content of primary cell cultures was determined by anti-EpCAM immunofluorescence (clone 4G10 Abcam, Heidelberg, Germany) and flow cytometry (9). The thymic carcinoma cell line 1889c was cultured under the same conditions. WNT4-overexpressing thymic epithelial cells (WNT4-TEP1) (32) were established using retroviral transgenesis and flow-cytometric enrichment as published previously (25).

### 3D multicellular spheroid and organoid-like culture of primary thymic epithelial cells

Freshly isolated pTECs were grown in two ways: To generate 3D multicellular spheroids (MCS), cells were seeded in ultra-low attachment 96-well plates (Corning, Germany) at 5 × 10^3^ cells/well in RPMI 1640 with 2 g/L glucose, 25 mM HEPES, 200 mM L-Glutamine, 50 U/ml penicillin, 50 µg/ml streptomycin and 10% calf serum (Sigma Aldrich, Germany). To generate organoid-like spheroids, 1 × 10^5^ cells were dispersed in 200 µl of ice-cold Matrigel (Corning, Germany), and then 30 µl of Matrigel drops were individually placed into pre-warmed flat-bottom 6-well plates. Plates were inverted, stored at 37°C for 40 min and then fed with the MEBM medium (Lonza) supplemented with B27 supplement (GIBCO), 0.5 μg/ml hydrocortisone (Sigma), 5 μg/ml insulin (Sigma), 4 μg/ml heparin (Sigma), 20 ng/ml bFGF (Invitrogen), and 20 ng/ml EGF (Sigma) (33). MCS and organoid-like spheres were collected for RNA and protein analysis. Imaging of 3D culture was performed using an inverse microscope, Zeiss Axio Observer Z1.

### Conditioned medium collection

Supernatants were obtained from pTECs cultured in RPMI 1640 with 2 g/L glucose, 25 mM HEPES, 200 mM L-Glutamine, 10% calf serum, and antibiotics for 96 h. Supernatants from the first cultures (p0, ‘early passage’) and subsequent passages (p1 to p5, ‘late passage’) were collected, centrifuged at 2,000*g* for 15 min, and stored at −80°C. Freeze-thaw cycles were kept to a minimum by aliquoting.

### pTEC cultures in conditioned medium

The MTT proliferation assay is based on the mitochondrial dehydrogenase activity as a surrogate proliferation marker. Stimulated pTECs were incubated with 10% (v:v) MTT (tetrazolium salt (3-(4,5-dimethylthiazol-2-yl)-2,5-diphenyltetrazolium bromide) for 2 h at 37°C. Subsequently, supernatants were discarded and the cells were lysed with DMSO. Dissolved pTECs, in which MTT was reduced to its insoluble formazan with purple, were quantified by measuring absorbance (570 nm and 600 nm) using a multi-well plate microreader TECAN (Infinite 200, Austria).

### Enzyme-linked immunosorbent assay

The WNT4 protein in thawed supernatants was measured by ELISA (Diagnostic System Laboratories, Webster, TX) following the instructions of the manufacturer. The assay employed an antibody-specific for human WNT4 coated on a 96-well plate. The WNT4 in calibration solutions and samples bound to the wells by the immobilized antibody were visualized by an HRP conjugated streptavidin detection system and measured with a TECAN ELISA reader at 450 nm. All protein lysate samples from cells and supernatants were assayed in triplicates. The mean of the three values was used for further calculation using standard values and GraphPad prism software for ELISA quantification.

### Immunoprecipitation and western blot

Before WNT4 immunoprecipitation (IP), cell culture supernatants were concentrated using trichloroacetic acid (20%) (TCA, Merck, Darmstadt, Germany) at a 1:1 dilution for protein precipitation. After two acetone washes, precipitated pellets were either suspended in 500 µl IP Lysis buffer (Pierce, Thermo Fisher Scientific, Germany) for subsequent IP or in 300 µl protein loading buffer (2× Laemmli buffer) and stored at −80°C for further protein analysis.

From the concentrated supernatant macromolecules stored in IP buffer, WNT4 was precipitated after overnight rotation at 4°C with 5 µg of anti-WNT4 antibody (Biozol Diagnostica, Germany) bound to 20 µl of protein A Sepharose according to the instructions of the manufacturer (GE Healthcare Germany). Briefly after incubation, the samples were centrifuged for 30 s at 4°C and 3,000×*g* and the pellet was washed five times with 500 µl of 1× cell lysis buffer. Finally, the pellet was resuspended with 20 μl of 1× SDS sample buffer, mixed by vortexing, then microcentrifuged for 30 s at 3,000×*g*, and the supernatant was subjected to Western blotting (9).

### Isolation of nuclear and cytoplasmic protein

The NE-PERTM Nuclear and Cytoplasmic Extraction kit was used to separate nuclear and cytoplasmic proteins according to the instructions of the manufacturer, followed by Western blot.

### Immunohistochemistry

Immunohistochemical detection of β-catenin protein expression *in situ* was performed in six B3 thymomas and nine thymic carcinoma samples using a rabbit monoclonal antibody against human β-catenin (Cell Signaling, CA, clone 8480s). Immunohistochemistry was performed using a Dako Autostainer (Dako Agilent, Germany). Briefly, 1 μm thick sections of formalin-fixed, paraffin-embedded material were deparaffinized using Xylene and rehydrated in graded ethanol concentrations. Antigen retrieval was performed with an epitope retrieval solution (Cell Signaling, CA) at pH 6.0 for 40 min and cooled down to room temperature for 30 min. Sections were then incubated with a 1:50 dilution in Antibody Diluent (Zytomed ZUCO-25-500) of β-catenin antibody after endogenous peroxidase blocking for 7 min. Sections were incubated with a peroxidase-labeled polymer conjugated to goat anti-mouse or goat anti-rabbit immunoglobulins as a secondary antibody for 30 min (Dako REAL™ EnVision™/HRP, Rabbit/Mouse (ENV)). Staining was visualized with 3, 3’-diaminobenzidine (DAB) as chromogen and slides were counterstained with hematoxylin, dehydrated, and finally mounted. Sections of desmoid tumors showing nuclear β-catenin expression served as a positive control.

### Short hairpin construction and cell transfection

Two WNT4 shRNA interference plasmids were constructed using the pU6-shRNA vector targeting the following sequences: GAAGAGGAAACTTAACCAC, GCAGACAAACCAAGAATGC. AB and B3 thymoma-derived pTECs and 1889c cells were transfected with WNT4-shRNA and control shRNA for 48 h using lipofectamine 2000 (Fischer Scientific, Germany) as described in a previous study (34).

### Real-time PCR analysis

Approximately 1–2 µg of RNA was isolated from whole tissues using TRIzol reagent (Invitrogen). Total RNA was reverse transcribed with RevertAid™ H Minus Reverse Transcriptase (Fermentas, Germany) using the protocol of the manufacturer. Real-time PCR was performed on the ABI STEP ONE PLUS TaqMan PCR System (Applied Biosystems, Germany) using FAST SYBR Green master mix (Applied Biosystems, Germany). The fold change in expression was calculated using the ΔΔCt method with GAPDH and cytokeratin 19 (CK19) as an internal control. Primer sequences are available in **Table S1**.

### Reagents

Lyophilized recombinant WNT4 protein (R&D Systems, Germany) was reconstituted at 50 μg/ml in PBS and stored at −80°C. The porcupine inhibitor IWP3 (Sigma-Aldrich, St. Louis, MO), AKT, and IKK inhibitors MK2206 and TPCA-1 and EF24 (3,5-Bis (2 flurobenzylidene) piperidine-4-one) (Selleckchem and Tocris, Germany) were dissolved in dimethyl sulfoxide (DMSO) (Sigma-Aldrich, St. Louis) at final stock concentrations of 10 mM, 15 mM, and 25 mM, respectively. Antibodies against β-catenin (Cell Signaling Technology, D10A8), WNT4 (9HCLC, Thermofisher), GAPDH (EPR16891, Abcam, USA), phospho-AKT (s473, D9E, Cell signaling Technology) and NF-κB p65 (D14E12, Cell Signaling Technology), phospho-IKKα/β (S176/180, 16A6), phospho-JUN antibodies (Sigma-Aldrich, St. Louis, MO), RAC1 antibody (2465, Cell signaling Technology) and β-actin (sc4778, Santa Cruz), anti-rabbit and anti-mouse peroxidase-conjugated secondary antibodies (Cell Signaling Technology) were used for Western blotting or immunohistochemistry.

### Statistical analyses

All statistical analyses were performed using GraphPad Prism V6.0 (GraphPad Software, Inc., La Jolla, USA). Two-tailed Student’s t-test and one-way ANOVA were applied when comparing WNT ligands and WNT frizzled receptor mRNA levels in different groups of thymomas. The subsequent Tukey’s multiple comparison test was used to compare variances among all the groups at a confidence level of 95%, with p <0.05 being considered as significant.

## Results

### B3 thymomas and TCs show increased WNT4 ligand and its frizzled receptor 6 (FZD6) expression

**Figure 1:**
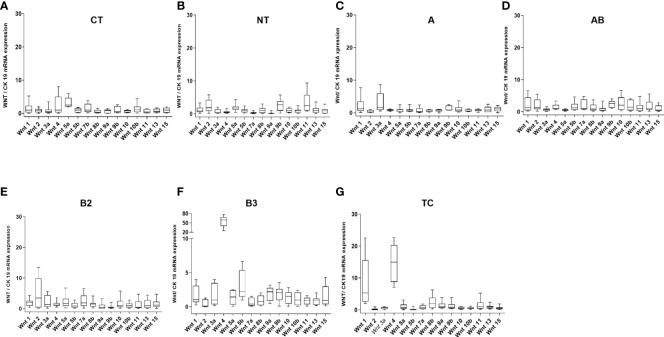
mRNA expression levels of 15 WNT ligands in non-neoplastic thymuses and thymic epithelial tumors as determined by Q-PCR. **(A)**: Childhood thymuses (CT) (n = 6); **(B)**: Normal thymuses (NT) (n = 15); **(C)**: Type A thymoma (n = 9); **(D)**: AB thymoma (n = 21); **(E)**: B2 thymoma (n = 13); **(F)**: B3 thymoma (n = 10) and **(G)**: Thymic carcinoma (TC, n = 10). Cytokeratin 19 was used as a reference to take the variable content of non-neoplastic thymocytes and WNT-producing thymic epithelial cells in the various thymomas into account.

**Figure 2:**
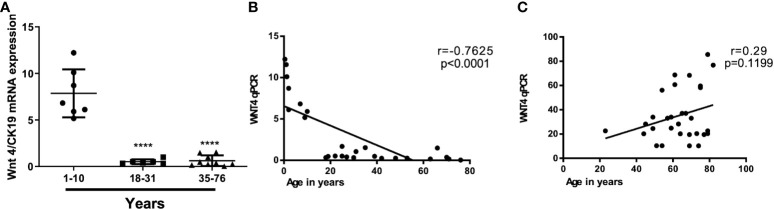
Age-independent WNT4 mRNA levels in thymic epithelial tumors (TETs) as compared to declining levels with age in non-neoplastic thymuses. **(A)**: *WNT4* mRNA expression by Q-PCR in 21 normal thymuses in different decades from childhood thymuses (n = 6) to adult normal thymuses (n = 15); **(B)**: Correlation between WNT4 expression levels and age in non-neoplastic thymuses (NT) and **(C)**: Correlation between WNT4 expression levels and age in B3 thymomas and thymic carcinomas (1). Pearson’s correlation (r) values are indicated within each graph.

**Figure 3:**
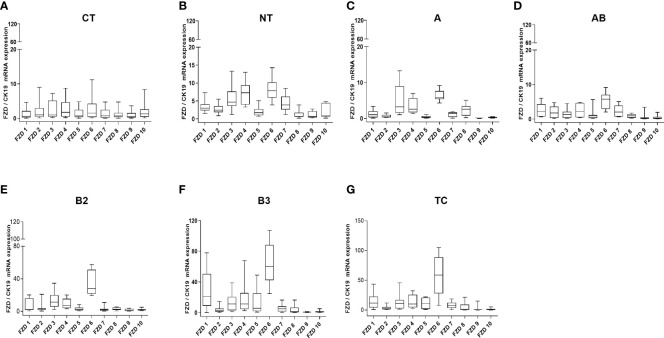
WNT frizzled receptor mRNA expression analysis. mRNA expression of 10 FZD receptors by Q-PCR in normal thymuses and TETs: **(A)**: 1 to 10 years old childhood thymuses (n = 6); **(B)**: adult thymuses (n = 15); **(C)**: thymoma type A (n = 9); **(D)**: thymoma type AB (n = 23); **(E)**: thymoma type B2 (n = 18); **(F)**: thymoma type 3 (n = 18) and **(G)**: thymic carcinoma (TC, n = 11). Cytokeratin 19 was used as reference gene to take the variable content of non-neoplastic thymocytes and WNT-producing thymic epithelial cells in the various thymomas into account.

At the mRNA level, the expression of most WNT genes was higher in B3 thymomas and TCs compared to other thymoma types and non-neoplastic thymuses (NT), when compared to epithelial marker CK19 expression (**Figure 1**). Results for WNT4 are detailed in **Table 2**. As shown in **Figure 2**, *WNT4* mRNA levels in NTs declined with age from childhood to the sixth decade, whereas no age-dependent decline in *WNT4* expression was observed in TETs. In addition to *WNT4*, the *WNT2* gene was strongly expressed in B2 thymomas compared with B3 thymomas and TCs (**Figure 1E**). All FZD receptors showed lower expression in NTs and indolent type A and AB thymomas compared with aggressive types B2 and B3 thymomas. High levels of *FZD6* mRNA were a recurrent finding in all aggressive TETs (B2, B3 thymomas, and TCs) (**Figure 3**). In contrast to *WNT4*, expression of FZD receptors showed no age-dependent differences in NT and thymic tumors, as shown in **Supplementary Figure 3**.

**Table 2:** Statistical values of the comparison of *WNT4* gene expression in thymic epithelial tumors (TETs), including thymomas and thymic carcinomas, with normal childhood thymuses (A, n = 6) and normal adults thymuses (B, n = 15) as represented in **Figure 1** (two-tailed Student’s *t*-test was applied for statistical analysis).

TETs vs. childhood thymuses	p-values	Significance		TETs vs. adult normal thymuses	p-values	Significance
A	0.0494	*		A	0.5270	ns
AB	<0.0001	****		AB	<0.0001	****
B2	0.007	***		B2	0.0016	**
B3	0.0021	**		B3	0.0021	****
TC	<0.0001	****		TC	<0.0001	****

*p <0.1, **p<0.01, ***p<0.001, ****p<0.0001. ns, not significant.

### Increased nuclear expression of WNT inhibitors TLE2 and TCF4 hampers nuclear β-catenin in B3 thymoma and TC

The levels of mRNA of selected endogenous inhibitors of the WNT pathway—membrane inhibitors (SFRP2, SFRP5, DKK1, and DKK4), cytosolic inhibitors (TIMP1–3), and nuclear inhibitors (TLE2, TLE4, TCF3, and TCF4) were quantified in 7 NTs and 62 TETs (8A, 25 AB, 12 B2, 7 B3, and 10 TC). *SFRP2* and *SFRP5* expression levels were increased in B2 thymomas, while AB thymomas showed increased *DKK1* and *DKK4* expression compared to other TETs and NTs (**Supplementary Figure 1**). The expression of cytosolic inhibitors, *TIMP1–3* was largely lacking in TETs and NTs (**Supplementary Figure 1**). In contrast, levels of nuclear inhibitors were variably higher in thymomas and TCs than in NTs: *TLE2* and *TCF4* were significantly (p <0.01) higher in B3 thymomas and TCs, *TLE4* only in B3 thymomas and *TCF3* only in AB thymomas (**Supplementary Figure 1**). Furthermore, β-catenin protein analysis by immunohistochemistry showed cytosolic staining in B3 thymomas and TC tissues (**Supplementary Figures 2C**, **D**) in contrast to the positive control (a desmoid tumor with β-catenin mutation), which showed ß-catenin nuclear staining (**Supplementary Figures 2A**, **B**) (35, 36).

### Conditioned medium from fresh thymoma-derived pTECs supports thymic epithelial cell survival

**Figure 4:**
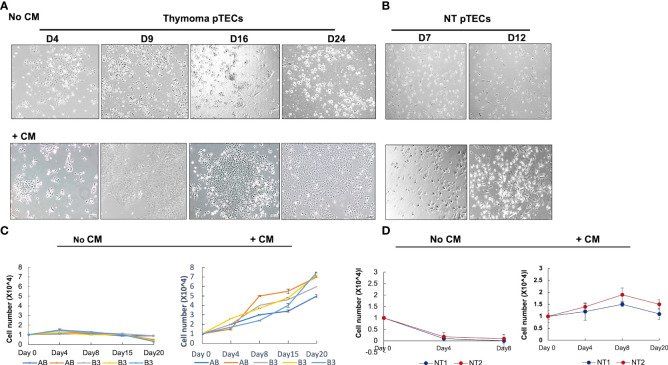
Effect of conditioned medium (CM) on primary thymic epithelial cells (pTECs) derived from thymomas and normal adult thymuses. Thymoma and normal thymus-derived pTECs were seeded in the presence of neoplastic thymic tumor conditioned media (CM) or the control standard medium (RPMI/Hepes/FBS) in 96-well plates. **(A)**: representative images of B3 thymoma-derived pTECs cultured from 4 to 24 days in the absence or presence of CM; **(B)**: representative images of normal thymus-derived pTECs from 7 to 12 days in the absence or presence of CM; **(C, D)**: Cell proliferation measured using MTT assay after culturing AB and B3 thymoma-derived pTECs **(C)** and NT-derived pTECs **(D)** with and without CM. No CM, without conditioned medium. Triplicate measurements of MTT were performed of two independent experiments. FBS, fetal bovine serum.

As mentioned above, WNT4 is secreted by normal thymic epithelial cells and activates a signaling network (25). Conditioned medium (CM) collected from pTECs culture was used for pTEC culturing at 25% v/v concentration and showed maintained cell morphology and allowed the expansion of thymoma-derived pTECs for 24 days (**Figures 4A**–**C**), but CM only maintained the viability of NT-derived pTECs without significant expansion for 8 days (**Figures 4B**–**D**). In contrast, the numbers of viable cells declined when pTECs from NT and thymomas were grown in standard RPMI/HEPES/FBS medium without conditioned medium (**Figures 4C**, **D**). Further, we wondered whether exogenous recombinant WNT4 ligand (rec-WNT4) or CM could rescue pTECs proliferation after WNT4 knockdown. Transfected using WNT4-shRNA for 48 h were 2 × 10^4^ of B3 thymoma-derived pTECs (n = 2) and were then further cultured and maintained for 7 days with either 100 ng/ml rec-WNT4 ligand or additional CM at a concentration of 25%v:v. As shown in **Supplementary Figure 4**, WNT4 knockdown decreased cell proliferation by nearly 65% and induced cell death, which was rescued by rec-WNT4 or CM-enriched media.

### WNT4 secretion increases in aggressive TETs and in ex vivo cultured thymoma epithelial cells

**Figure 5:**
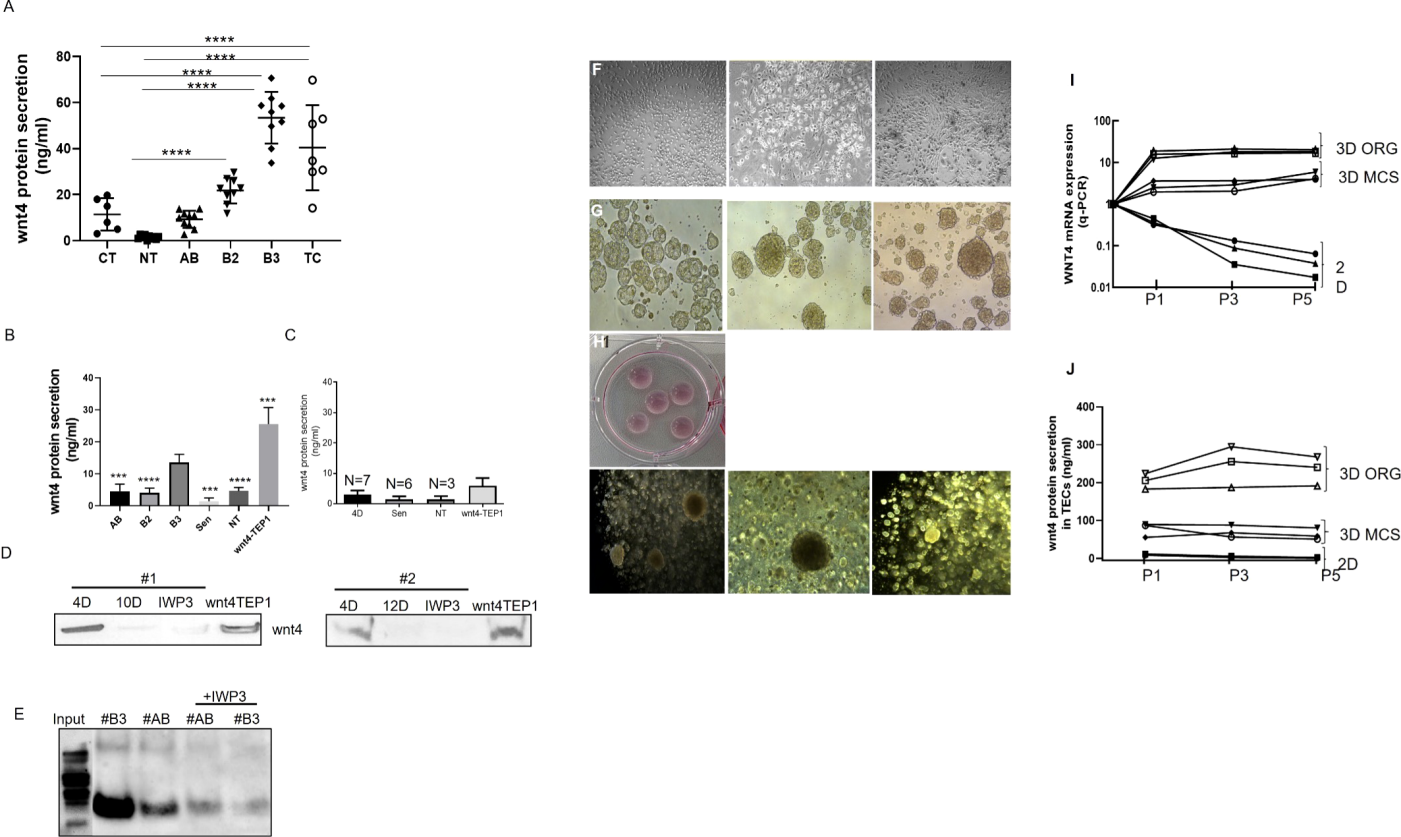
WNT4 secretion analysis *in situ* in normal and tumor tissues and in primary thymic epithelial cells (pTECs) in 2D and 3D cell culture models. **(A)**: ELISA-based measurement of WNT4 in protein lysates from whole tissues of 6 childhood and 10 aged normal thymuses and thymic tumors of 10 AB, 10 B2, 10 B3, and 8 TC; **(B)**: Supernatants of pTECs were measured by ELISA from 3 NT, 8 AB, 3 B2, and 7 B3 thymomas and from long-term cultures of 3 AB and 3 B3 thymoma-derived pTECs. WNT4-TEP1 cells are murine thymic epithelial cells transfected with a human WNT4 expression vector (positive control); **(C)**: ELISA measurement of WNT4 in supernatants of pTECs from 3 NT, 3 AB, and 4 B3 treated for 3 days with the WNT secretion inhibitor, IWP3; **(D)**: Western blot-based determination of WNT4 in cell culture supernatants collected from 2 B3 cases (#1, #2) after short-term (4-day, 4D) and long-term (10- or 12-day, 10D, 12D) culture with or without treatment of IWP3. Supernatants from WNT4-TEP1 cells served as a positive control. Protein was concentrated by trichloroacetic acid precipitation (20%, 1:1); **(E)**: Immunoprecipitation of WNT4 from supernatants collected from short-term pTECs cultures of an AB and B3 thymoma with or without IWP3 treatment. Prior to immunoprecipitation, cell culture supernatants were concentrated using Amicon filtration; **(F)**: Microscopic images of 2D monolayer cell cultures of primary TECs (1 AB and 2 B3); **(G)** Microscopic images of pTECs from the same thymomas cultured in 3D multicellular spheroid (MCS, Matrigel-free) for 4 days; **(H)**: Representative images of 3D organoid (ORG, Matrigel+) cell culture; **(I)**: WNT4 mRNA expression analysis (Q-PCR) of 3 pTECs (1AB and 2B3) cultured in 2D and 3D (MCS and organoid) for 3 passages (P1(5days) P3 (14 days) and P5 (20 days)); **(J)**: Wnt4 secretion quantification in cell culture supernatants in three different passages by ELISA. Images were taken with a LEICA microscope with ×200 for 2D and ×100 for 3D cultures. D, days, Sen, Senescent. ***p<0.001, ****p<0.0001.

WNT4 secretion was evaluated in whole tissues of normal thymuses (six childhood and 10 aged NT) and thymic tumors (10 AB, 10B2 and 10B3 and 8TC) and was significantly higher in B3 thymomas and TCs compared with childhood and aged non-neoplastic thymuses (**Figure 5A**). Further WNT4 protein secretion from 4-day-old *ex vivo* cultured (‘fresh’) pTECs was significantly higher in B3-pTECs (n = 7) than NT-pTECs (n = 3), AB-pTECs (n = 8) and B2-pTECs (n = 3) (****p <0.0001, ***p = 0.0003) when measured by ELISA in cell culture supernatants (‘conditioned medium’). WNT4 secretion from fresh B3-pTECs was significantly higher than secretion after 10–20 days of culture (****p <0.0001; n = 6) (**Figures 5B**, **C**). When fresh pTECs and WNT4/TEP1 cells were treated with the WNT secretion inhibitor IWP3, WNT4 release was reduced by 75% (**Figures 5B**–**E**). WNT4 secretion by TEP1 cells was distinctly higher than that by fresh B3-pTECs.

### pTECs grown in 3D maintained WNT4 expression and secretion dependent on an autocrine loop

We have shown the positive effect of WNT4-containing conditioned medium on proliferation and expansion of pTECs in standard 2D cultures (**Figure 4**), but whether WNT4 itself can also drive WNT4 expression in pTECs *in vivo* is a mystery.

To address this hypothesis, tymoma-derived pTECs were grown as 2D and 3D spheroids (with and without Matrigel) (**Figures 5G, H**) with the basic medium. As shown in **Figures 5I, J**, pTECs cultured in 3D models prevented the time-dependent decline of WNT4 mRNA expression in thymoma-derived pTECs, suggesting the operation of an autocrine loop in the 3D settings. Interestingly, WNT4 mRNA expression was higher in Matrigel-covered than Matrigel-free 3D pTEC cultures, suggesting an impact of Matrigel on WNT4 expression and secretion. Furthermore, the expression of the JNK/JUN target genes, *FOXN1*, *MYC*, and *CD44* was also driven by CM-enriched or recombinant WNT4-supplemented media, but more so by growth in 3D (**Supplementary Figure 4**).

### WNT4 activates the non-canonical WNT/JNK pathway in thymoma-derived pTECs

Since the absence of nuclear β-catenin suggested a minor role of canonical WNT signaling in TETs (**Supplementary Figure 2**), the role of the non-canonical WNT/JNK pathway in TETs and its correlation with WNT4 is still unknown. Both WNT4 and RAC1 expression and JNK phosphorylation were maintained in 2D pTECs when cultured in WNT4-containing conditioned medium or in recombinant WNT4-enriched media compared to pTECs cultured in standard media, which showed a decline of those proteins in long-term cultures. WNT4 protein expression was higher in B3-pTECs than AB-pTECs (**Supplementary Figure 5**).

### Crosstalk between WNT4/PCP, AKT, and NF-κB pathways in thymoma-derived pTECs

**Figure 6:**
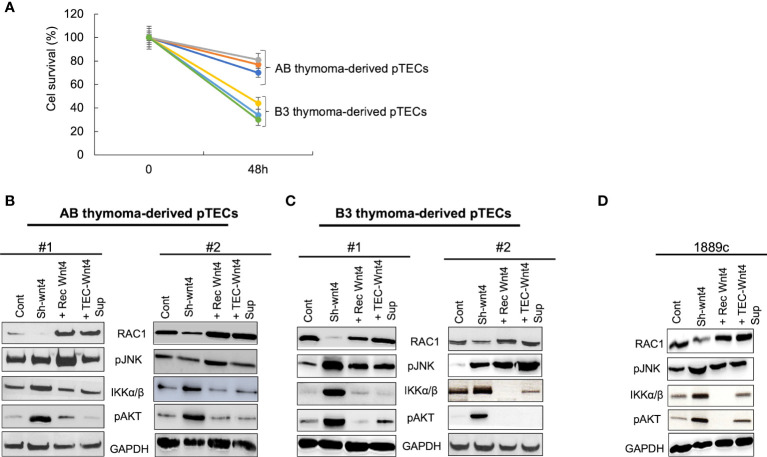
WNT4 knockdown decreased cell proliferation and RAC1 protein but not pJNK in early pTECs. Thymoma-derived pTECs and thymic carcinoma 1889c cell line were either transfected using pU6WNT4-shRNA or stimulated with either 200 ng/ml recombinant WNT4 or with the CM 25% (v:v) (Wnt4 Sup). Proteins of RAC1, pJNK, IKKα/β, and pAKT were extracted from the cells and analyzed by western blot after transfection of 48 h or after four days of stimulation. **(A)**: Cell proliferation analysis in AB and B3 thymoma derived pTECs (n = 6) after transfection for 48 h with pU6-WNT4-shRNA. Cell proliferation was measured using MTT assay; **(B)**: Protein analysis of RAC1, pJNK, IKKα/β, pAKT, in pTECs derived from 2 cases of AB thymomas; **(C)**: Protein analysis of RAC1, pJNK, IKKα/β, pAKT, in pTECs derived from two cases of B3 thymomas; **(D)**: Protein analysis of RAC1, pJNK, IKKα/β, pAKT in thymic carcinoma cell line, 1889c. GAPDH is used as loading control for cytosolic protein fractions.

To elucidate whether the gradual decline of WNT4, RAC1, and pJNK expression in long-term 2D pTEC cultures is caused by declining WNT4 expression, we knocked down the *WNT4* gene in pTECs in early passages of culture using shRNA, which led to reduced proliferation of AB-pTECs (n = 3), but particularly more of B3-pTECs (n = 3) (**Figure 6A**), and decreased RAC1 protein expression, which could be rescued by exogenous WNT4 (**Figures 6B**–**D**). Unexpectedly, decreasing RAC1 levels in *WNT4* KD were accompanied by increasing pJNK levels, although JNK is a target of RAC1 (37, 38) (**Figures 6B**–**D**). Thus, the short-term knockdown of WNT4 in early stages of pTECs did not mimic the spontaneous decline of WNT4, RAC1, and pJNK during long-term 2D pTEC cultures in WNT4-deficient growth medium, as shown in **Supplementary Figure 5**.

**Figure 7:**
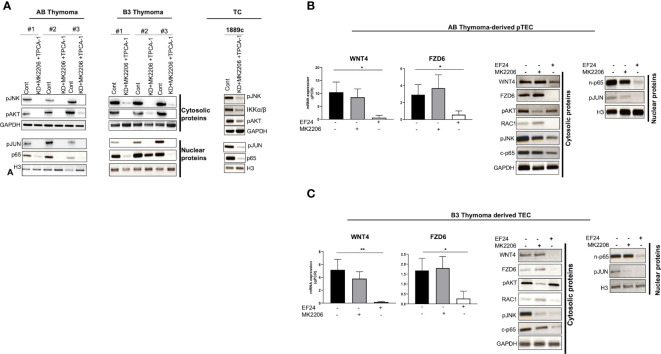
NF-κB activated non-canonical WNT4/PCP pathway. **(A)**: pJNK inactivation and down-regulation of pAKT and NF-κB (p65) in AB and B3 thymoma-derived pTECs (n = 3 each) and thymic carcinoma 1889c cell line, which were first transfected with pU6WNT4-shRNA, then treated with MK2206 and TPCA1 that target pAKT and IKKα/β (NF-κB pathway), respectively; **(B, C)**: pJNK down regulation by targeting p65 (NF-κB) or pAKT in AB and B3 thymoma-derived pTECs (n = 1 each); pTECs were treated either with 1 µM EF24 or 2 µM MK2206 for 48 h that target p65 (NF-κB) and pAKT respectively. WNT4 and FZD6 mRNA levels were quantified using Q-PCR. Cytosolic and nuclear proteins were isolated then analyzed by western blot for the following molecules: WNT4, FZD6, RAC1, pJNK, pAKT, p65, and pJUN. GAPDH and H3 were used as loading control. Cont, either with sh-wnt4 transfected nor drug treated cells; c-p65, Cytosolic p65; n-p65, Nuclear p65. *p<0.1, **p<0.01.

To understand this paradox, we next investigated the expression of pAKT and pIKKα/β, since activated AKT and NF-κB pathways can induce JNK phosphorylation in other cell types (39, 40). Indeed, *WNT4* KD strongly increased levels of pAKT and pIKK in neoplastic pTECs and 1889c cells (**Figures 6B**–**D**), which was accompanied by stable pJNK levels in AB-pTECs (**Figure 6B**) or even increased pJNK levels in B3-pTECs and 1889c cells (**Figures 6C**, **D**). This suggests a crosstalk of the non-canonical WNT4/PCP pathway with AKT and NF-κB pathways in pTECs that maintained high levels of pJNK following *acute* deficiency of WNT4. Since *WNT4* KD increased JNK, AKT, and IKKα/β phosphorylation, we asked the question whether *WNT4* KD could be prevented through the combination of AKT and pIKKα/β inhibitors MK2206 and TPCA1. Indeed JNK phosphorylation was completely inhibited in the presence of the combinations (*WNT4* KD, MK2206, and TPCA1) (**Figure 7A**).

### NF-κB signaling is an upstream activator of the WNT4/PCP pathway in pTECs

Since *WNT4* KD increased pJNK, pAKT, and pIKKα/β phosphorylation, we asked the questions of whether AKT and NF-κB pathways could act upstream of the non-canonical WNT4/PCP pathway in pTECs. Pharmacological inhibition of AKT using MK2206 or NF-κB directly targeting p65 with EF24 affected negatively the non-canonical WNT4/PCP pathway only in EF24 treated pTECs. Both mRNA and protein levels of WNT4 and FZD6 and their downstream proteins RAC1 and pJNK were decreased, and the nuclear translocation of target pJUN was prevented (**Figures 7B**, **C**). In contrast, pAKT inhibition with MK2206 showed no such effects.

## Discussion

**Figure 8:**
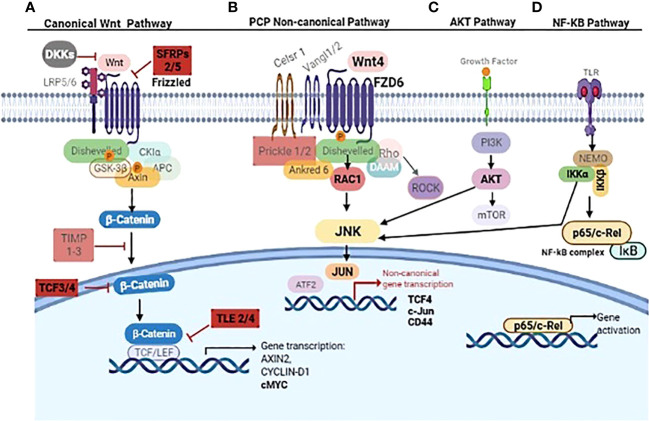
Overview of non-canonical WNT4/NF-κB/AKT signaling cascades in thymoma subtype B3 and thymic carcinoma. The canonical ß-catenin depending WNT pathway inhibitors SFRP2/5, TLE2/4, and TCF 3/4 are mentioned in **(A)**. WNT4/FZD6 leads to PCP non-canonical pathway activation through RAC1/JNK **(B)**. IKK complex (IKKα/β) and AKT phosphorylate JNK linking NF-kB and AKT signaling to non-canonical Wnt4 pathway **(C, D)**. Key molecules WNT4, FZD6, SFRP, TCF3/4, TLE2/4, RAC1, JNK, JUN, IKKα/β, AKT, p65, CD44, and cMYC are in bold. Other molecules in gray are either not relevant in thymoma and thymic carcinoma or still unknown in these tumors. The figure was created using BioRender.com software.

New findings here were i) WNT4 overexpression in aggressive B3 thymomas and TCs compared to thymomas (A, AB, and B2) and NTs contrary to Chen et al. (41), who showed WNT4 overexpression in all thymoma types; ii) expression of WNT frizzled receptor 6 (*FZD6*) is stronger in B3 thymomas and TCs compared to other thymomas and NTs; iii) the absence of physiological, age-related decline of *WNT4* expression in TETs; iv) a WNT4-driven autocrine loop that activates WNT/PCP/JNK pathway in 3D-cultured neoplastic pTECs; and v) stabilization of JNK activation in neoplastic pTECs through ‘compensatory’ activation of AKT and NF-κB pathways following acute blockade of WNT4 signaling. These findings are summarized in an overview in **Figure 8**.

### Relevance of epithelial WNT4/FZD6 overexpression and secretion in TETs

Previous studies have shown that declining *WNT4* and *FOXN1* expression levels are critically involved in thymic senescence, i.e., physiological shrinkage of the thymic parenchyma with age (26, 42, 43). Furthermore, over-expressed *WNT4* and *FOXN1* (42) and preliminary data from an AB thymoma cell line suggest that increased *WNT4* expression plays a role in thymoma development through non-canonical WNT signaling (41). These findings inspired our working hypothesis that interference with thymic senescence could contribute to oncogenesis across the spectrum of thymomas and TCs through the abnormal expression of *WNT4* (23, 44, 45). Furthermore, increased *WNT4* levels in TETs did not show any correlation with the age of patients, suggesting physiological senescence signals regulating WNT4 expression in the normal thymus are muted in TETs (**Figure 2**). An overexpression of WNT4 frizzled receptor *FZD6* accompanied the increase in *WNT4* (**Figure 3**). This is a key new finding that WNT4/FZD6 appears to be particularly relevant for developing the most aggressive TETs (B3 thymomas and TCs) since they showed the most abnormally increased mRNA levels of *WNT4/FZD6.*

In contrast, inhibitors of canonical WNT signaling, *TLE2/4* and *TCF4*, were strongly expressed (**Supplementary Figure 1**), while *nuclear* β-catenin was undetectable in tissues of aggressive TETs *in situ* (**Supplementary Figure 2**). Therefore, abnormal activation of non-canonical WNT4/PCP but non-canonical WNT pathway appears to play a major role in the pathogenesis of aggressive TETs. This conclusion fits well with the observations in mice, in which neither TEC-specific ablation nor abnormal activation of the canonical WNT pathway elicited thymic epithelial neoplasms but structurally abnormal and atrophic thymuses with compromised thymopoiesis (26, 46, 47). Although abnormal activation of the canonical WNT pathway and autocrine WNT/β-catenin loops (48, 49) are of utmost pathogenetic relevance in many cancers (e.g., colorectal cancer) (50, 51), activated non-canonical WNT pathways can drive oncogenesis as well (52). Non-canonical WNT-driven oncogenesis, however, typically involves WNT5a, WNT7, and WNT11 (52) while WNT4 plays a role in only a few carcinoma types, e.g., hepatocellular carcinomas (53) and a breast cancer subset (1, 54). Interestingly, *WNT4* expression in these cancers is accompanied by increased levels of *FZD6* and *MYC*, i.e., features that we detected in aggressive TETs previously (4). However, a WNT4-driven autocrine loop has not been described so far in another cancer type to our knowledge and may be one more unique molecular peculiarity of TETs (10, 55).

The conclusion of WNT4 expression and secretion in the most aggressive TETs (B3 thymomas and TCs) is strongly underpinned by our *in vitro* experiments, since the rich conditioned medium could postpone senescence, maintain proliferation, and extend the survival of neoplastic pTECs grown *in vitro* (**Figure 4**). In line with this peculiarity of TETs, induced WNT4 expression in thymoma-derived pTECs was positively correlated with the expression of *FOXN1* (**Supplementary Figure 4**). This suggests that WNT4 drives expression of *FOXN1*, i.e., the master regulator of thymus development and maintenance in neoplastic pTECs in a similar way as in the normal thymus (56), including the rare quiescent but non-senescent TECs that presumably represent TEC progenitors in adult thymuses (57). These cells can resume proliferation after acute insults (e.g., chemotherapy) to support thymus regeneration and form clusters in a particular niche at the cortico-medullary junction, suggesting that cell–cell contact may be required to maintaining their Wnt4^high^Foxn1^high^ phenotype (57). Since we report that neoplastic pTECs in 3D culture maintain the WNT4^+^FOXN1^+^ phenotype of TETs much longer than in 2D cultures, we hypothesize that tumor cell–tumor cell and tumor cell–matrix interactions may be important to maintain the autocrine WNT4 loop, *FOXN1* expression, and tumor cell survival of TETs *in vivo*. In support of the critical relevance of cell–cell contacts in this scenario, even the invasion front of TETs typically shows cohesive tumor cells, and the establishment of representative TET cell lines in 2D cultures has been exceptionally unsuccessful (55).

It is unknown which molecules mediate the clustering of the Wnt4^high^Foxn1^high^ presumed TEC progenitors in normal adult murine thymuses (57) and the cell–cell and cell–matrix interactions in human TETs. As for cell–matrix interactions, *CD44* appears as a possible candidate since it is expressed by neoplastic pTECs preferentially in 3D cultures (**Supplementary Figure 4**) as well as in aggressive TETs *in vivo* (58) and—together with *WNT4*—is a marker of cancer stem cells (59–61). As for cell–cell interactions, identifying respective molecules in TETs should be a research priority since interference with the 3D structure of neoplastic TECs might elicit senescence through attenuation of the WNT4–FOXN1 axis and thus have therapeutic potential in TET patients. Since the treatment of pTECs with exogenous WNT4 in 2D cultures induced the transcription of the *WNT4* gene (**Supplementary Figure 4**), WNT4 transcription and secretion are more induced in 3D (MCS and ORG) than in 2D cultures of neoplastic pTECs (**Figures 5I**, **J**). It has also been reported that WNT4 levels were increased in the serum of CRC patients and originated from CRC tissues (62). Further, it appears that an autocrine WNT4 loop is operative in TETs *in vitro*. Apparently, this abnormality is an intrinsic property of the tumor cells, since 3D cultures of neoplastic TECs showed stronger WNT4 expression and secretion (**Figures 5I**, **J**) for longer periods than parallel 2D neoplastic TEC cultures from the same tumors.

The WNT4-driven expression of JNK in a stable AB thymoma cell line has been reported previously (41, 44) and conforms to our finding that the WNT4 autocrine loop depends on WNT4-driven RAC1 and JNK expression, i.e., activation of the non-canonical WNT/PCP pathway (52) in neoplastic pTECs derived mainly from aggressive TETs (**Supplementary Figure 5**). Finally, RAC1 and JNK could be detected in B3 thymoma and TC tissues as reported by Chen et al. with JNK in terms of JNK expression (41).

### Link of NF-κB to WNT4 non-canonical pathway in thymoma derived pTECs

A new finding here is that acute WNT4 blockade in pTECs induces activation of NF-κB and AKT, which in turn, maintains or even increases levels of phosphorylated JNK. This has not been reported previously. In fact, this new observation of WNT/NF-κB crosstalk in pTECs appears to be at odds with previous reports in cancers (63, 64), in which NF-κB activation inhibited JNK phosphorylation.

JNK is not only involved in non-canonical WNT signaling but also in the mitogen-activated protein kinase (MAPK) cascade, which plays a vital role in apoptosis, proliferation, migration, survival and tumorigenesis (65). Some published studies suggest that WNT/AKT-mTOR signaling is an important step in controlling cancer cell metabolism (66). JNK phosphorylation in the WNT4/PCP pathway is not only dependent on WNT4/FZD6 but also on NF-κB signaling in TECs (**Figure 7**). However, NF-κB activation either shuts down JNK or inhibits TNFα-induced apoptotic signaling (67) or blocks JNK-dependent cell death by counteracting reactive oxygen species (ROS) accumulation, which in turn triggers JNK activation (64) and WNT/AKT. These signaling pathways can modulate the downstream target gene transcriptional activation of the WNT signaling pathway, providing insight into a potential molecular mechanism for inflammation-induced carcinogenesis (68). In our study, both NF-κB and AKT activated JNK, but but only NF-κB blockade WNT4/FZD6/JNK signaling using EF-24. (**Figure 7**). Our results suggest that WNT4 signaling drives thymoma oncogenesis and that acute blocking of WNT4 signaling—under a therapeutic perspective—might induce resistance through AKT/NF-κB activation.

### Translational perspective

Considering that the most aggressive TETs, B3 thymomas, and TCs showed combined increased expression of WNT4, its non-canonical frizzled receptor 6 (FZD6) (41, 44), and downstream non-canonical WNT/JNK activity, TETs appear as promising candidates for trials testing novel inhibitors of WNT and JNK (52). Indeed, targeting WNT signaling (canonical or non-canonical, which are the currently addressed targets), small molecular inhibitors and antibodies have already been effective against cancers *in vitro* (69) and monoclonal antibodies against FZD receptors are currently being tested in clinical trials NCT01973309 against breast cancers and NCT01957007 against solid tumors or NCT01957007 in synergy with docetaxel against colorectal adenocarcinoma as well as NCT02005315 with paclitaxel and gemcitabine targeting metastatic pancreatic ductal adenocarcinoma (70). WNT secretion is also targeted by some inhibitors like the inhibitor IWP-2 for treatment of cancers, especially pancreatic cancers with RNF43 mutations (69, 71, 72), whereas PORCN inhibitors WNT974 and ETC-159 are applicable to prevent mammary tumors and cancer stem cells (73–75). However, from our observation that WNT4 blockade *in vitro* activated pro-survival AKT and NF-κB signaling in neoplastic pTECs, we anticipate that monotherapies targeting WNT signaling alone might be insufficient for targeting TETs. Instead, the combined blockade of WNT, AKT, and NF-κB signaling, as shown here *in vitro*, should be considered to be tested against aggressive TETs *in vivo*. Considering our new observations, WNT4 is also expressed by thymocytes and may foster tumor cells in a paracrine way. Targeting thymocytes (e.g., by corticosteroids) may complement tumor cell-directed interventions and may interact with immune cells in pre-clinical models of cancer using porcupine inhibitors (76).

## Data availability statement

The original contributions presented in the study are included in the article/**supplementary material**. Further inquiries can be directed to the corresponding author.

## Ethics statement

The studies involving human participants were reviewed and approved by the Ethik-Kommission II der University Heidelberg Medicine Faculty Mannheim (#2009-290N-MA/2010 and 2018-516N-MA). Written informed consent to participate in this study was provided by the participants’ legal guardian/next of kin.

## Author contributions

The study was conceived and designed by DB and AM. The experiments were conducted by DB and XZ. Clinical data and material support were provided by BS, KK, MK, TG, GO, KK, EB, PS and ML. WMT4 over-expressing cells were established by KK. Data were analyzed by DB and XZ. The manuscript was written by DB, AM, and XZ and DB was the coordinator and made a major revision of the whole work. All authors have read and agreed to the published version of the manuscript

## Funding

KK received funding for this collaborative research from the National Research, Development and Innovation Fund of Hungary, financed under the TKP2021-NVA funding scheme.

## Acknowledgments

The authors would like to express deep appreciation to all the patients who provided their samples for this study and all the collaborators from the hospitals of Heidelberg, Stuttgart and Regensburg. The author XZ, is financially supported by the China Scholarship Council. We would like to express sincere thanks to AG, BS, RR, DK, PJ, PH, KW, TG, GO, PG, and ML who provided us the clinical data, material, and technical support.

## Conflict of interest

The authors declare that the research was conducted in the absence of any commercial or financial relationships that could be construed as a potential conflict of interest.

## Publisher’s note

All claims expressed in this article are solely those of the authors and do not necessarily represent those of their affiliated organizations, or those of the publisher, the editors and the reviewers. Any product that may be evaluated in this article, or claim that may be made by its manufacturer, is not guaranteed or endorsed by the publisher.

## References

[1] Vouyovitch CM Perry JK Liu DX Bezin L Vilain E Diaz JJ. WNT4 mediates the autocrine effects of growth hormone in mammary carcinoma cells. Endocr Relat Cancer (2016) 23(7):571–85. doi: 10.1530/ERC-15-0528 27323961

[2] Strobel P Hohenberger P Marx A. Thymoma and thymic carcinoma: molecular pathology and targeted therapy. J Thorac Oncol (2010) 5(10 Suppl 4):S286–90. doi: 10.1097/JTO.0b013e3181f209a8 20859121

[3] Board., W.C.o.T.E. Thoracic tumours. Lyon (France): International agency for research on cancer. In: WHO classification of tumours series, 5(5th ed (2021).

[4] Huang Belharazem BD Li L Kneitz S Schnabel PA Rieker RJ. Anti-apoptotic signature in thymic squamous cell carcinomas - functional relevance of anti-apoptotic BIRC3 expression in the thymic carcinoma cell line 1889c. Front Oncol (2013) 3:16. doi: 10.3389/fonc.2013.00316 24427739 PMC3876280

[5] Petrini I Meltzer P Zucali P Luo J Lee C Santoro A. Copy number aberrations of BCL2 and CDKN2A/B identified by array-CGH in thymic epithelial tumors. Cell Death Dis (2012) 3:e351. doi: 10.1038/cddis.2012.92 22825469 PMC3406591

[6] Marx A CChan JK Chalabreysse L Dacic S Detterbeck F French CA. The 2021 WHO classification of tumors of the thymus and mediastinum: What is new in thymic epithelial, germ cell, and mesenchymal tumors? J Thorac Oncol (2022) 17(2):200–13. doi: 10.1016/j.jtho.2021.10.010 34695605

[7] Enkner F Pichlhöfer B Zaharie AT Krunic M Holper TM Janik S. Molecular profiling of thymoma and thymic carcinoma: Genetic differences and potential novel therapeutic targets. Pathol Oncol Res (2017) 23(3):551–64. doi: 10.1007/s12253-016-0144-8 PMC5487866 27844328

[8] Ahmad U Yao X Detterbeck F Huang J Antonicelli A Filosso PL. Thymic carcinoma outcomes and prognosis: results of an international analysis. J Thorac Cardiovasc Surg (2015) 149(1):95–100, 101.e1-2. doi: 10.1016/j.jtcvs.2014.09.124 25524678

[9] Belharazem D Grass A Paul C Vitacolonna M Schalke B Rieker RJ. Increased cFLIP expression in thymic epithelial tumors blocks autophagy via NF-kappaB signalling. Oncotarget (2017) 8(52):89580–94. doi: 10.18632/oncotarget.15929 PMC5685693 29163772

[10] Radovich M Pickering CR Felau I Ha G Zhang H Jo H. The integrated genomic landscape of thymic epithelial tumors. Cancer Cell (2018) 33(2):244–258.e10. doi: 10.1016/j.ccell.2018.01.003 29438696 PMC5994906

[11] Bleul CC Boehm T. BMP signaling is required for normal thymus development. J Immunol (2005) 175(8):5213–21. doi: 10.4049/jimmunol.175.8.5213 16210626

[12] Anderson G Pickering CR Felau I Ha G Zhang H Jo H. Notch ligand-bearing thymic epithelial cells initiate and sustain notch signaling in thymocytes independently of T cell receptor signaling. Eur J Immunol (2001) 31(11):3349–54. doi: 10.1002/1521-4141(200111)31:11<3349::AID-IMMU3349>3.0.CO;2-S 11745352

[13] Osada M Ito E Fermin HA Vazquez-Cintron E Venkatesh T Friedel RH. The wnt signaling antagonist Kremen1 is required for development of thymic architecture. Clin Dev Immunol (2006) 13(2-4):299–319. doi: 10.1080/17402520600935097 17162372 PMC2270768

[14] Huang C Ma R Xu Y Li N Li Z Yue J. Wnt2 promotes non-small cell lung cancer progression by activating WNT/beta-catenin pathway. Am J Cancer Res (2015) 5(3):1032–46. PMC4449433 26045984

[15] MacMillan CD Leong HS Dales DW Robertson AE Lewis JD Chambers AF. Stage of breast cancer progression influences cellular response to activation of the WNT/planar cell polarity pathway. Sci Rep (2014) 4:6315. doi: 10.1038/srep06315 25204426 PMC4159636

[16] Mulligan KA Fuerer C Ching W Fish M Willert K Nusse R. Secreted wingless-interacting molecule (Swim) promotes long-range signaling by maintaining wingless solubility. Proc Natl Acad Sci USA (2012) 109(2):370–7. doi: 10.1073/pnas.1119197109 PMC3258625 22203956

[17] Mlodzik M. Planar cell polarization: do the same mechanisms regulate drosophila tissue polarity and vertebrate gastrulation? Trends Genet (2002) 18(11):564–71. doi: 10.1016/S0168-9525(02)02770-1 12414186

[18] Jung YS Park JI. Wnt signaling in cancer: therapeutic targeting of wnt signaling beyond beta-catenin and the destruction complex. Exp Mol Med (2020) 52(2):183–91. doi: 10.1038/s12276-020-0380-6 PMC7062731 32037398

[19] Swann JB Happe C Boehm T. Elevated levels of wnt signaling disrupt thymus morphogenesis and function. Sci Rep (2017) 7(1):785. doi: 10.1038/s41598-017-00842-0 28400578 PMC5429746

[20] Komiya Y Habas R. Wnt signal transduction pathways. Organogenesis (2008) 4(2):68–75. doi: 10.4161/org.4.2.5851 19279717 PMC2634250

[21] Wei T Zhang N Guo Z Chi F Song Y Zhu X. Wnt4 signaling is associated with the decrease of proliferation and increase of apoptosis during age-related thymic involution. Mol Med Rep (2015) 12(5):7568–76. doi: 10.3892/mmr.2015.4343 26397044

[22] Heinonen KM Vanegas J Brochu S Shan J Seppo V Perreault C. Wnt4 regulates thymic cellularity through the expansion of thymic epithelial cells and early thymic progenitors. Blood (2011) 118(19):5163–73. doi: 10.1182/blood-2011-04-350553 21937690

[23] Varecza Z Kvell K Talabér G Miskei G Csongei V Bartis D. Multiple suppression pathways of canonical wnt signalling control thymic epithelial senescence. Mech Ageing Dev (2011) 132(5):249–56. doi: 10.1016/j.mad.2011.04.007 PMC3146701 21549744

[24] van Loosdregt J Fleskens V Tiemessen MM Mokry M van Boxtel R Meerding J. Canonical wnt signaling negatively modulates regulatory T cell function. Immunity (2013) 39(2):298–310. doi: 10.1016/j.immuni.2013.07.019 23954131

[25] Kvell K Varecza Z Bartis D Hesse S Parnell S Anderson G. Wnt4 and LAP2alpha as pacemakers of thymic epithelial senescence. PloS One (2010) 5(5):e10701. doi: 10.1371/journal.pone.0010701 20502698 PMC2872673

[26] Zuklys S Gill J KellerM P Hauri-Hohl M Zhanybekova S Balciunaite G. Stabilized beta-catenin in thymic epithelial cells blocks thymus development and function. J Immunol (2009) 182(5):2997–3007. doi: 10.4049/jimmunol.0713723 19234195

[27] Osada M Jardine L Misir R Andl T Millar SE Pezzano MD\. DKK1 mediated inhibition of wnt signaling in postnatal mice leads to loss of TEC progenitors and thymic degeneration. PloS One (2010) 5(2):e9062. doi: 10.1371/journal.pone.0009062 20161711 PMC2817005

[28] Pongracz J Hare K Harman B Anderson G Jenkinson EJ. Thymic epithelial cells provide WNT signals to developing thymocytes. Eur J Immunol (2003) 33(7):1949–56. doi: 10.1002/eji.200323564 12884861

[29] Ferrando-Martínez S Ruiz-Mateos E Dudakov JA Velardi E Grillari J Kreil DP. WNT signaling suppression in the senescent human thymus. J Gerontol A Biol Sci Med Sci (2015) 70(3):273–81. doi: 10.1093/gerona/glu030 PMC4351388 24657825

[30] van Andel H Kocemba K.A Spaargaren M Pals S. Aberrant wnt signaling in multiple myeloma: molecular mechanisms and targeting options. Leukemia (2019) 33(5):1063–75. doi: 10.1038/s41375-019-0404-1 PMC6756057 30770859

[31] Travis WD Brambilla E Burke AP Marx A Nicholson AG. Introduction to the 2015 world health organization classification of tumors of the lung, pleura, thymus, and heart. J Thorac Oncol (2015) 10(9):1240–2. doi: 10.1097/JTO.0000000000000663 26291007

[32] Beardsley TR Pierschbacher M Wetzel GD Hays EF. Induction of T-cell maturation by a cloned line of thymic epithelium (TEPI). Proc Natl Acad Sci USA (1983) 80(19):6005–9. doi: 10.1073/pnas.80.19.6005 PMC534348 6604278

[33] Ucar A Ucar O Klug P Matt S Brunk F Hofmann TG. Adult thymus contains FoxN1(-) epithelial stem cells that are bipotent for medullary and cortical thymic epithelial lineages. Immunity (2014) 41(2):257–69. doi: 10.1016/j.immuni.2014.07.005 PMC4148705 25148026

[34] Mosmann T. Rapid colorimetric assay for cellular growth and survival: application to proliferation and cytotoxicity assays. J Immunol Methods (1983) 65(1-2):55–63. doi: 10.1016/0022-1759(83)90303-4 6606682

[35] Mullen JT DeLaney TF Rosenberg AE Le L Iafrate AJ Kobayashi W. Beta-catenin mutation status and outcomes in sporadic desmoid tumors. Oncologist (2013) 18(9):1043–9. doi: 10.1634/theoncologist.2012-0449 PMC3780636 23960186

[36] Trautmann M Rehkämper J Gevensleben H Becker J Wardelmann E Hartmann W. Novel pathogenic alterations in pediatric and adult desmoid-type fibromatosis - a systematic analysis of 204 cases. Sci Rep (2020) 10(1):3368. doi: 10.1038/s41598-020-60237-6 32099073 PMC7042250

[37] Kim GH Han JK. JNK and ROKalpha function in the noncanonical Wnt/RhoA signaling pathway to regulate xenopus convergent extension movements. Dev Dyn (2005) 232(4):958–68. doi: 10.1002/dvdy.20262 15739222

[38] Yoshida T Zhang Y Rivera Rosado LA Chen J Khan T Moon SY. Blockade of Rac1 activity induces G1 cell cycle arrest or apoptosis in breast cancer cells through downregulation of cyclin D1, survivin, and X-linked inhibitor of apoptosis protein. Mol Cancer Ther (2010) 9(6):1657–68. doi: 10.1158/1535-7163.MCT-09-0906 20515940

[39] Liu J Lin A. Wiring the cell signaling circuitry by the NF-kappa b and JNK1 crosstalk and its applications in human diseases. Oncogene (2007) 26(22):3267–78. doi: 10.1038/sj.onc.1210417 17496921

[40] Choi Y Ko YS Park J Choi Y Kim Y Pyo JS. HER2-induced metastasis is mediated by AKT/JNK/EMT signaling pathway in gastric cancer. World J Gastroenterol (2016) 22(41):9141–53. doi: 10.3748/wjg.v22.i41.9141 PMC5107595 27895401

[41] Chen Y Zhang P Tang P Lv P Li X Wang Y. Wnt4 overexpression promotes thymoma development through a JNK-mediated planar cell polarity-like pathway. Oncol Lett (2018) 15(1):83–90. 29387212 10.3892/ol.2017.7266 PMC5769365

[42] Brembeck FH Rosario M Birchmeier W. Balancing cell adhesion and wnt signaling, the key role of beta-catenin. Curr Opin Genet Dev (2006) 16(1):51–9. doi: 10.1016/j.gde.2005.12.007 16377174

[43] Niehrs C. The complex world of WNT receptor signalling. Nat Rev Mol Cell Biol (2012) 13(12):767–79. doi: 10.1038/nrm3470 23151663

[44] Chen Y Liu X Liu Y Wang Y Wang H Lu C. Decreased Wnt4 expression inhibits thymoma development through downregulation of FoxN1. J Thorac Dis (2017) 9(6):1574–83. doi: 10.21037/jtd.2017.05.28 PMC5506175 28740671

[45] Talaber G Kvell K Varecza Z Boldizsar F Parnell SM Jenkinson EJ. Wnt-4 protects thymic epithelial cells against dexamethasone-induced senescence. Rejuv Res (2011) 14(3):241–8. doi: 10.1089/rej.2010.1110 PMC3136744 21453014

[46] Liang CC You LR Yen JJ Liao NS Yang-Yen HF Chen CM. Thymic epithelial beta-catenin is required for adult thymic homeostasis and function. Immunol Cell Biol (2013) 91(8):511–23. doi: 10.1038/icb.2013.34 23856765

[47] Kvell K Fejes AV Parnell SM Pongracz JE. Active wnt/beta-catenin signaling is required for embryonic thymic epithelial development and functionality ex vivo. Immunobiology (2014) 219(8):644–52. doi: 10.1016/j.imbio.2014.03.017 24768153

[48] Becker J Wilting J. WNT signaling in neuroblastoma. Cancers (Basel) (2019) 11(7). doi: 10.3390/cancers11071013 PMC6679057 31331081

[49] Akiri G Cherian MM Vijayakumar S Liu G Bafico A Aaronson SA. Wnt pathway aberrations including autocrine wnt activation occur at high frequency in human non-small-cell lung carcinoma. Oncogene (2009) 28(21):2163–72. doi: 10.1038/onc.2009.82 PMC4451819 19377513

[50] Lecarpentier Y Schussler O Hébert J-L Vallée A. Multiple targets of the canonical WNT/beta-catenin signaling in cancers. Front Oncol (2019) 9:1248. doi: 10.3389/fonc.2019.01248 31803621 PMC6876670

[51] Nusse R Clevers H. Wnt/beta-catenin signaling, disease, and emerging therapeutic modalities. Cell (2017) 169(6):985–99. doi: 10.1016/j.cell.2017.05.016 28575679

[52] Zhan T Rindtorff N Boutros M. Wnt signaling in cancer. Oncogene (2017) 36(11):1461–73. doi: 10.1038/onc.2016.304 PMC5357762 27617575

[53] Bengochea A de Souza MM Lefrançois L Le Roux E Galy O Chemin I. Common dysregulation of Wnt/Frizzled receptor elements in human hepatocellular carcinoma. Br J Cancer (2008) 99(1):143–50. doi: 10.1038/sj.bjc.6604422 PMC2453022 18577996

[54] Sikora MJ Jacobsen BM Levine K Chen J Davidson NE Lee AV. WNT4 mediates estrogen receptor signaling and endocrine resistance in invasive lobular carcinoma cell lines. Breast Cancer Res (2016) 18(1):92. doi: 10.1186/s13058-016-0748-7 27650553 PMC5028957

[55] Yamada Y Simon-Keller K Belharazem-Vitacolonnna D Bohnenberger H Kriegsmann M Kriegsmann K. A tuft cell-like signature is highly prevalent in thymic squamous cell carcinoma and delineates new molecular subsets among the major lung cancer histotypes. J Thorac Oncol (2021) 16(6):1003–1016. doi: 10.1016/j.jtho.2021.02.008 33609752

[56] Balciunaite G Keller MP Balciunaite E Piali L Zuklys S Mathieu YD. Wnt glycoproteins regulate the expression of FoxN1, the gene defective in nude mice. Nat Immunol (2002) 3(11):1102–8. doi: 10.1038/ni850 12379851

[57] Dumont-Lagacé M Brochu S St-Pierre C Perreault C. Adult thymic epithelium contains nonsenescent label-retaining cells. J Immunol (2014) 192(5):2219–26. doi: 10.4049/jimmunol.1302961 24477909

[58] Sonobe S Miyamoto H Nobukawa B Izumi H Futagawa T Ishikawa N. Prognostic value of CD44 isoform expression in thymic epithelial neoplasms. Cancer (2005) 103(10):2015–22. doi: 10.1002/cncr.21046 15830350

[59] Russo A Colina JA Moy J Baligod S Czarnecki AA Varughese P. Silencing PTEN in the fallopian tube promotes enrichment of cancer stem cell-like function through loss of PAX2. Cell Death Dis (2021) 12(4):375. doi: 10.1038/s41419-021-03663-2 33828085 PMC8027874

[60] Katoh M. Canonical and non-canonical WNT signaling in cancer stem cells and their niches: Cellular heterogeneity, omics reprogramming, targeted therapy and tumor plasticity (Review). Int J Oncol (2017) 51(5):1357–69. doi: 10.3892/ijo.2017.4129 PMC5642388 29048660

[61] Liu S Yin P Dotts AJ Kujawa SA Coon VJS Wei JJ. Activation of protein kinase b by WNT4 as a regulator of uterine leiomyoma stem cell function. Fertil Steril (2020) 114(6):1339–49. doi: 10.1016/j.fertnstert.2020.06.045 PMC7722234 32892998

[62] Yang D Li Q Shang R Yao L Wu L Zhang M. WNT4 secreted by tumor tissues promotes tumor progression in colorectal cancer by activation of the wnt/beta-catenin signalling pathway. J Exp Clin Cancer Res (2020) 39(1):251. 33222684 10.1186/s13046-020-01774-w PMC7682076

[63] Tornatore L Sandomenico A Raimondo D Low C Rocci A Tralau-Stewart C. Cancer-selective targeting of the NF-kappaB survival pathway with GADD45beta/MKK7 inhibitors. Cancer Cell (2014) 26(6):938. 10.1016/j.ccell.2014.11.021 PMC5629036 28898681

[64] Verzella D Pescatore A Capece D Vecchiotti D Ursini MV Franzoso G. Life, death, and autophagy in cancer: NF-kappaB turns up everywhere. Cell Death Dis (2020) 11(3):210. doi: 10.1038/s41419-020-2399-y 32231206 PMC7105474

[65] Xu R Hu J. The role of JNK in prostate cancer progression and therapeutic strategies. BioMed Pharmacother (2020) 121:109679. doi: 10.1016/j.biopha.2019.109679 31810118

[66] Vadlakonda L Pasupuleti M Pallu R. Role of PI3K-AKT-mTOR and wnt signaling pathways in transition of G1-s phase of cell cycle in cancer cells. Front Oncol (2013) 3:85. doi: 10.3389/fonc.2013.00085 23596569 PMC3624606

[67] Franzoso G Zazzeroni F Papa S. JNK: a killer on a transcriptional leash. Cell Death Differ (2003) 10(1):13–5. doi: 10.1038/sj.cdd.4401154 12655290

[68] Anderson EC Wong MH. Caught in the akt: regulation of wnt signaling in the intestine. Gastroenterology (2010) 139(3):718–22. doi: 10.1053/j.gastro.2010.07.012 PMC3037729 20659460

[69] Steinhart Z Pavlovic Z Chandrashekhar M Hart T Wang X Zhang X. Genome-wide CRISPR screens reveal a wnt-FZD5 signaling circuit as a druggable vulnerability of RNF43-mutant pancreatic tumors. Nat Med (2017) 23(1):60–8. doi: 10.1038/nm.4219 27869803

[70] Kim MJ Huang Y Park JI. Targeting wnt signaling for gastrointestinal cancer therapy: Present and evolving views. Cancers (Basel) (2020) 12(12). doi: 10.3390/cancers12123638 PMC7761926 33291655

[71] Steinhart Z Pavlovic Z Chandrashekhar M Hart T Wang X Zhang X. Corrigendum: Genome-wide CRISPR screens reveal a wnt-FZD5 signaling circuit as a druggable vulnerability of RNF43-mutant pancreatic tumors. Nat Med (2017) 23(11):1384. doi: 10.1038/nm1117-1384d 29117169

[72] Valenta T Degirmenci B Moor AE Herr P Zimmerli D Moor MB. Wnt ligands secreted by subepithelial mesenchymal cells are essential for the survival of intestinal stem cells and gut homeostasis. Cell Rep (2016) 15(5):911–8. doi: 10.1016/j.celrep.2016.03.088 27117411

[73] Tammela T Sanchez-Rivera FJ Cetinbas NM Wu K Joshi NS Helenius K. A wnt-producing niche drives proliferative potential and progression in lung adenocarcinoma. Nature (2017) 545(7654):355–9. doi: 10.1038/nature22334 PMC5903678 28489818

[74] Chen B Dodge ME Tang W Lu J Ma Z Fan CW. Small molecule-mediated disruption of wnt-dependent signaling in tissue regeneration and cancer. Nat Chem Biol (2009) 5(2):100–7. doi: 10.1038/nchembio.137 PMC2628455 19125156

[75] Proffitt KD Madan B Ke Z Pendharkar V Ding L Lee MA. Pharmacological inhibition of the wnt acyltransferase PORCN prevents growth of WNT-driven mammary cancer. Cancer Res (2013) 73(2):502–7. doi: 10.1158/0008-5472.CAN-12-2258 23188502

[76] Haseeb M Pirzada RH Ain QU Choi SW. Wnt signaling in the regulation of immune cell and cancer therapeutics. Cells (2019) 8(11). doi: 10.3390/cells8111380 PMC6912555 31684152

