# Supplementary material for: WNT4 overexpression and secretion in thymic epithelial tumors drive an autocrine loop in tumor cells in vitro
Source: Front Oncol. 2022 Jul 29;12:920871. doi: 10.3389/fonc.2022.920871 (PMC9372913; doi:10.3389/fonc.2022.920871)
Supplement: Supplementary file 1 [file DataSheet_1.docx]

Supplementary Material

# Supplementary Figures and Tables

## Supplementary Figures

**
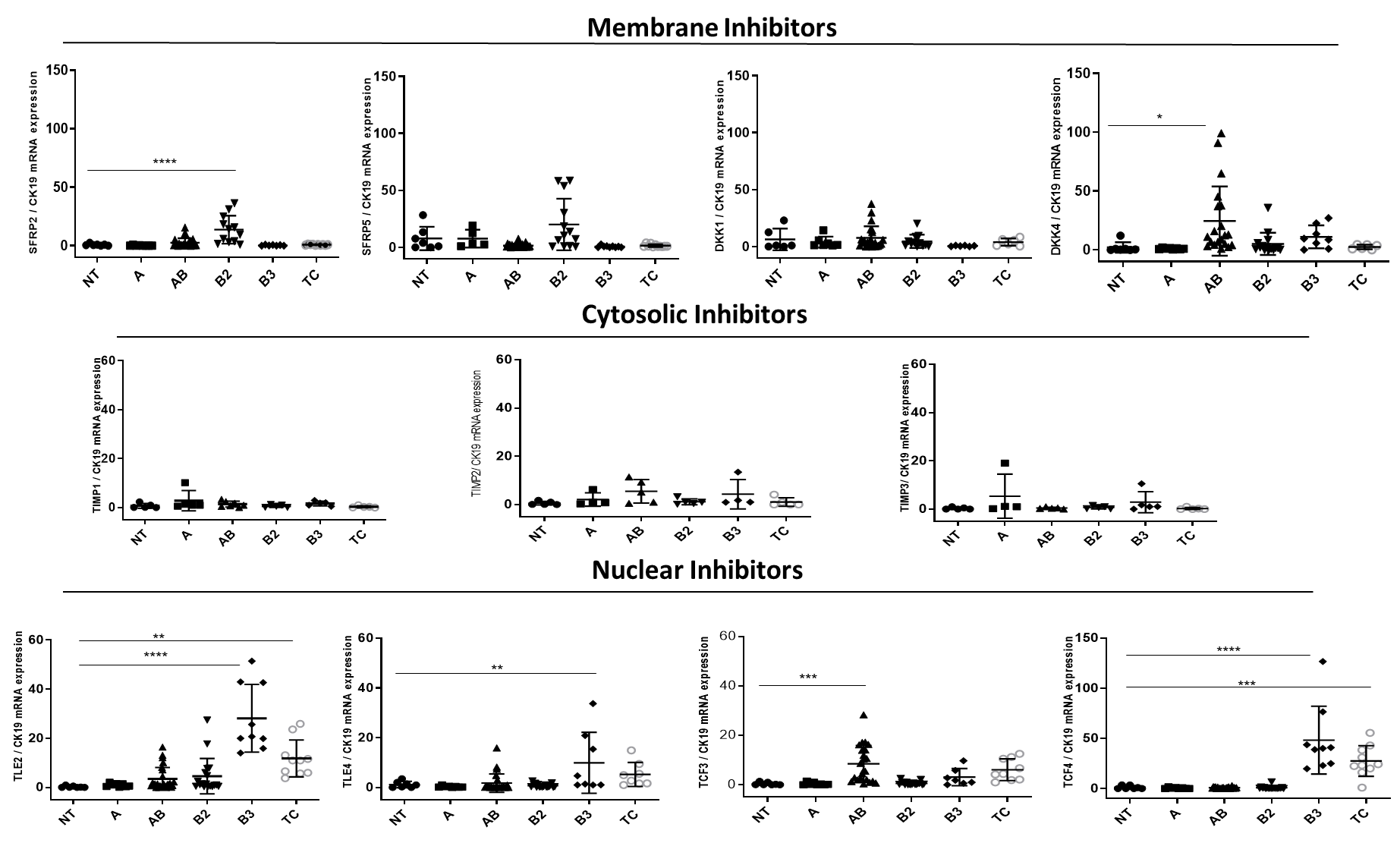
**

**Supplementary Figure 1. mRNA quantification of WNT inhibitors in thymic tumors and adult normal thymuses (NT).** Expression levels of WNT signaling inhibitors in thymomas (n=60) and thymic carcinomas (n=11) compared to NT (n=7). Cytokeratin 19 was used as endogen reference for the Q-PCR analysis. ****:p<0.0001. ***: p=0.0007 and 0.0004, **: p= 0.0048 and 0.0034, *: p=0.0232

**
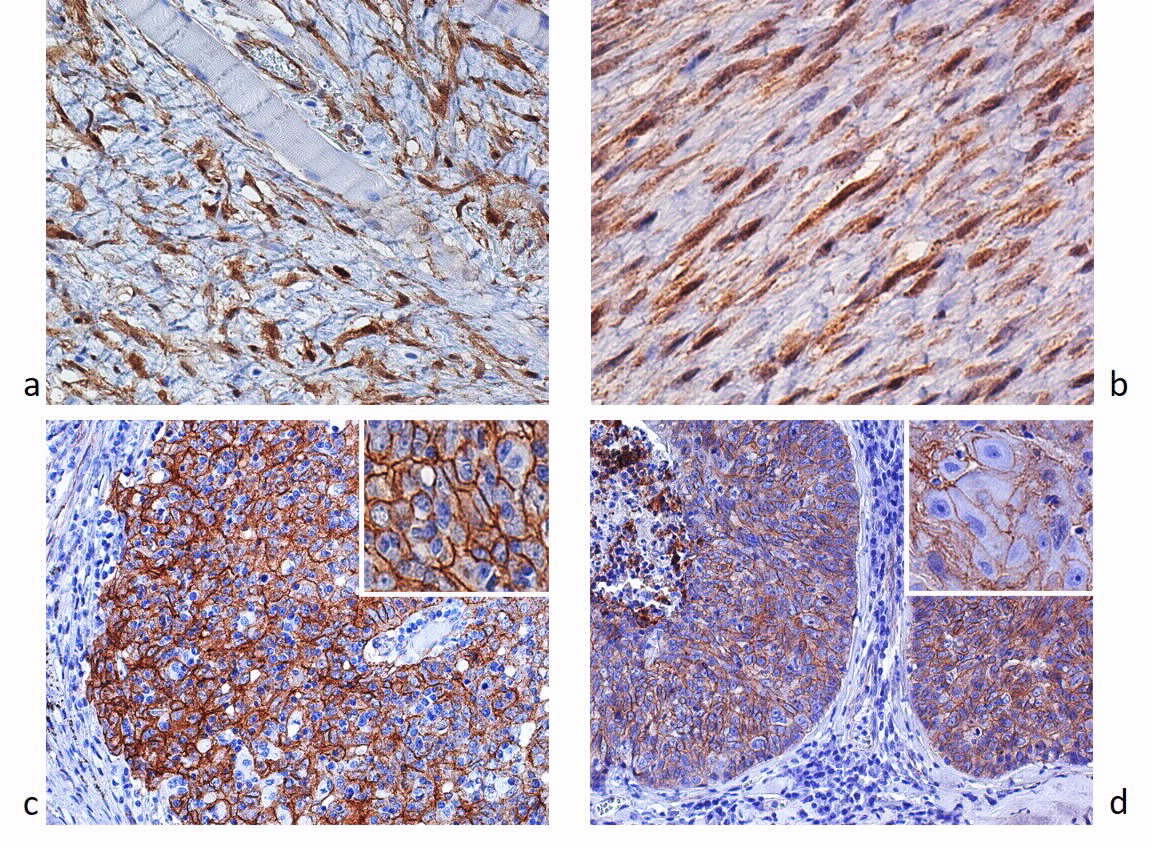
**

**Supplementary Figure 2.** **Expression of the ß-catenin protein in B3 thymoma and thymic carcinoma.** (**A**, **B**): Immunostaining of cytoplasmic and nuclear ß-catenin in desmoid fibromatosis (control, A: x200, B: x400); (**C**): ß-catenin immunostaining mainly of the cell membrane in a representative B3 thymoma (x200; inset x400); (**D**): ß-catenin immunostaining mainly of the cell membrane in a representative TSQCC (x200; inset 400). A-D: immune peroxidase.


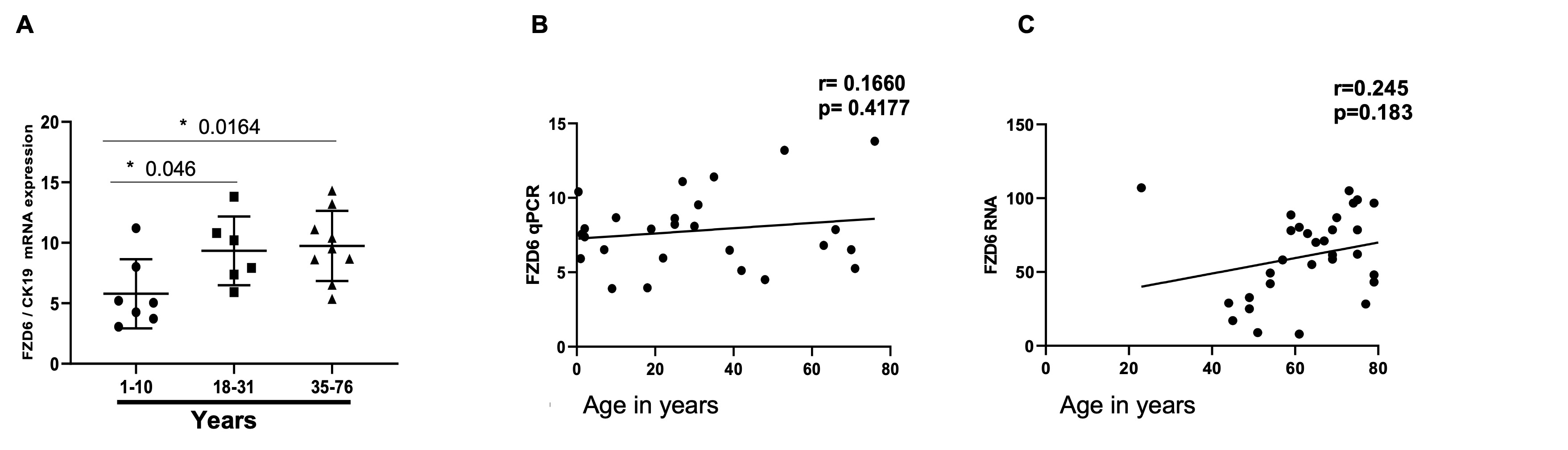


**Supplementary Figure 3:**  Age-independent FZD6 mRNA levels in thymic epithelial tumors (TETs) as compared to slightly increased levels with age in non-neoplastic thymuses. (A): FZD6 mRNA expression by Q-PCR in 21 normal thymuses in different decades from childhood thymuses (n=7) to adult normal thymuses (n=15); (B): Correlation between FZD6 expression levels and age in non-neoplastic thymuses (NT) and (C): Correlation between FZD6 expression levels and age in B3 thymomas and thymic carcinomas. Pearson's correlation (r) values are indicated within each graph. CK19 gene is used as intern control to take the variable content of non-neoplastic thymocytes in the various thymomas into account since there's no CK19 gene expression in thymocytes.

*
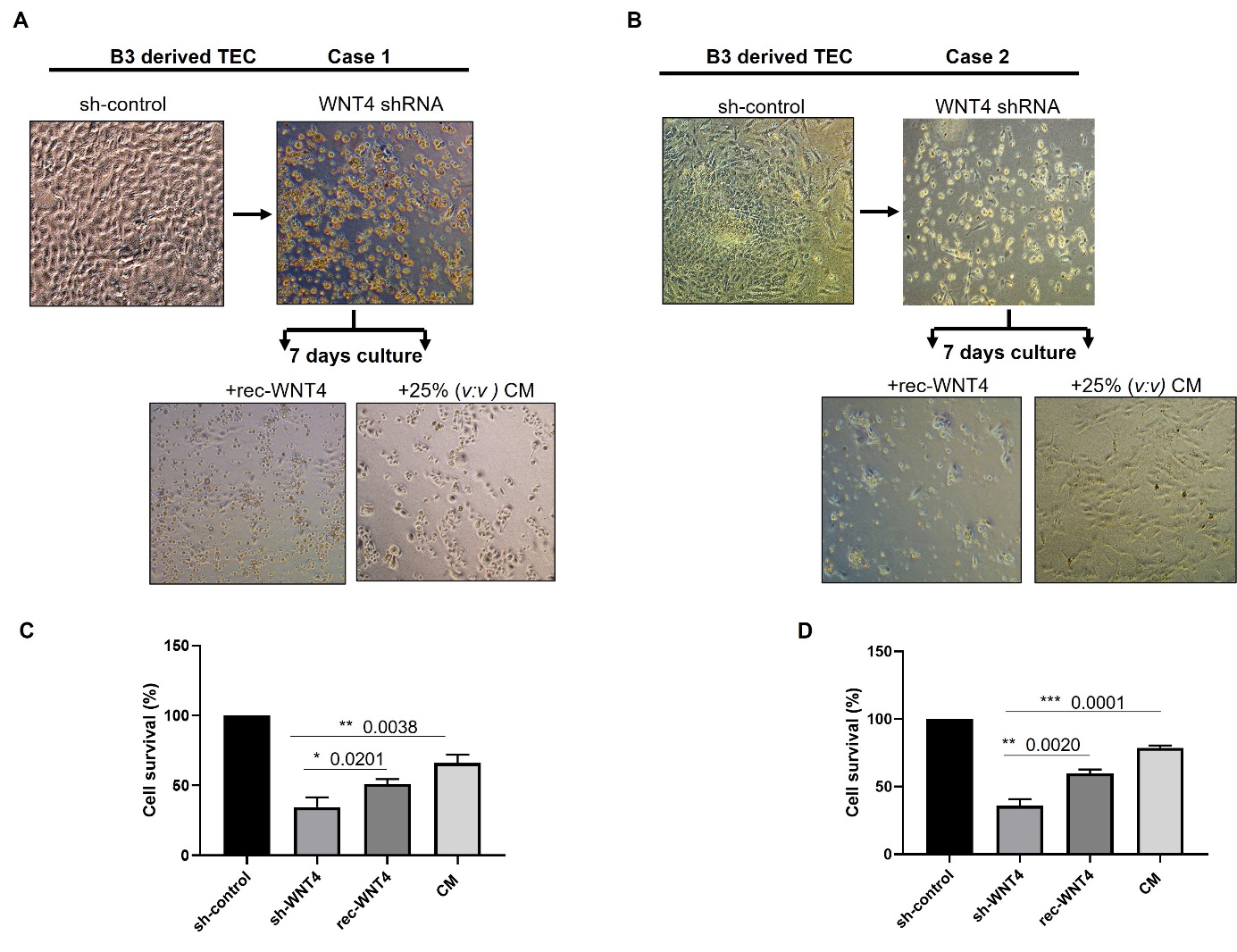
*

**Supplementary Figure 4**: Effect of exogenous rec-WNT4 and conditioned medium (CM) on primary thymic epithelial cells (pTECs) derived from B3 thymomas. 2X10^4^ cells /well of B3 thymoma-derived pTECs were transfected using WNT4-shRNA in 96 well plates for 48h and further cultured for 7days using 100ng/ml rec-WNT4 ligand or CM enriched media. (A): representative images of B3 thymoma-derived pTECs before/ after WNT4 knockdown in presence of rec-WNT4 or CM. (B): Cell proliferation measured using MTT assay after WNT4 knockdown and further culturing B3 thymoma-derived pTECs with rec-WNT4 or CM. CM: conditioned medium. Triplicate measurements of MTT were performed of two independent experiments.


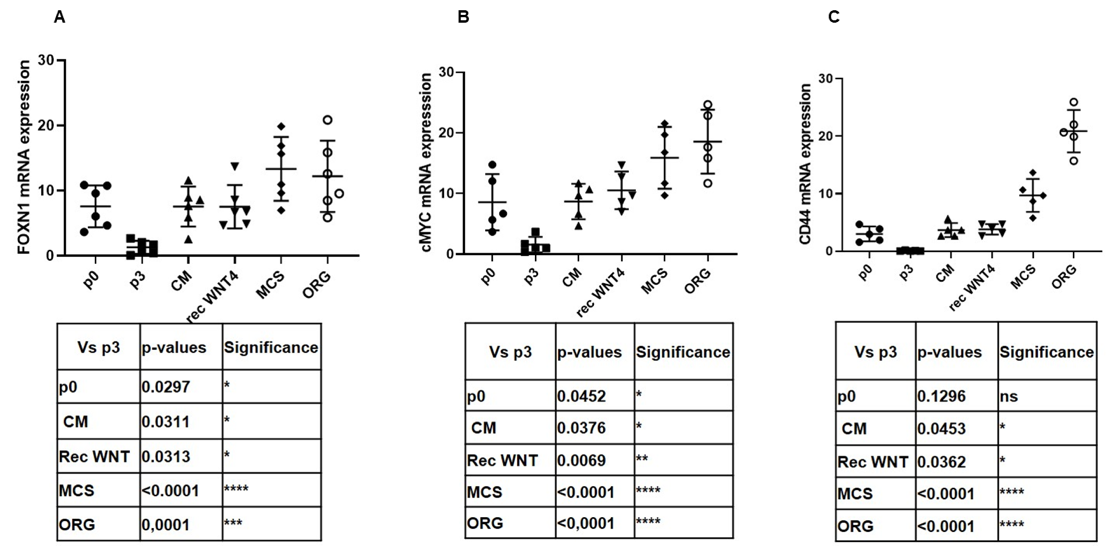


**Supplementary Figure 5.** **Q-PCR-based quantification of mRNA encoded by the pJNK/JUN dependent genes**. pJNK/JUN dependent genes *FOXN1*, *MYC* and *CD44* were quantified on mRNA level in pTECs (n=6: 3AB and 1B2 and 2B3) after short-term (p0), long-term (p3), conditioned media (CM)-enriched, and recombinant (rec) WNT4-enriched 2D cultures, as well as culture as 3D multicellular spheroid (MCS) and organoid (ORG) cultures. The tables present the p-values and statistical significances from the comparison of all cultures against p3 senescent cultures using the ANOVA test; ns: not significant.


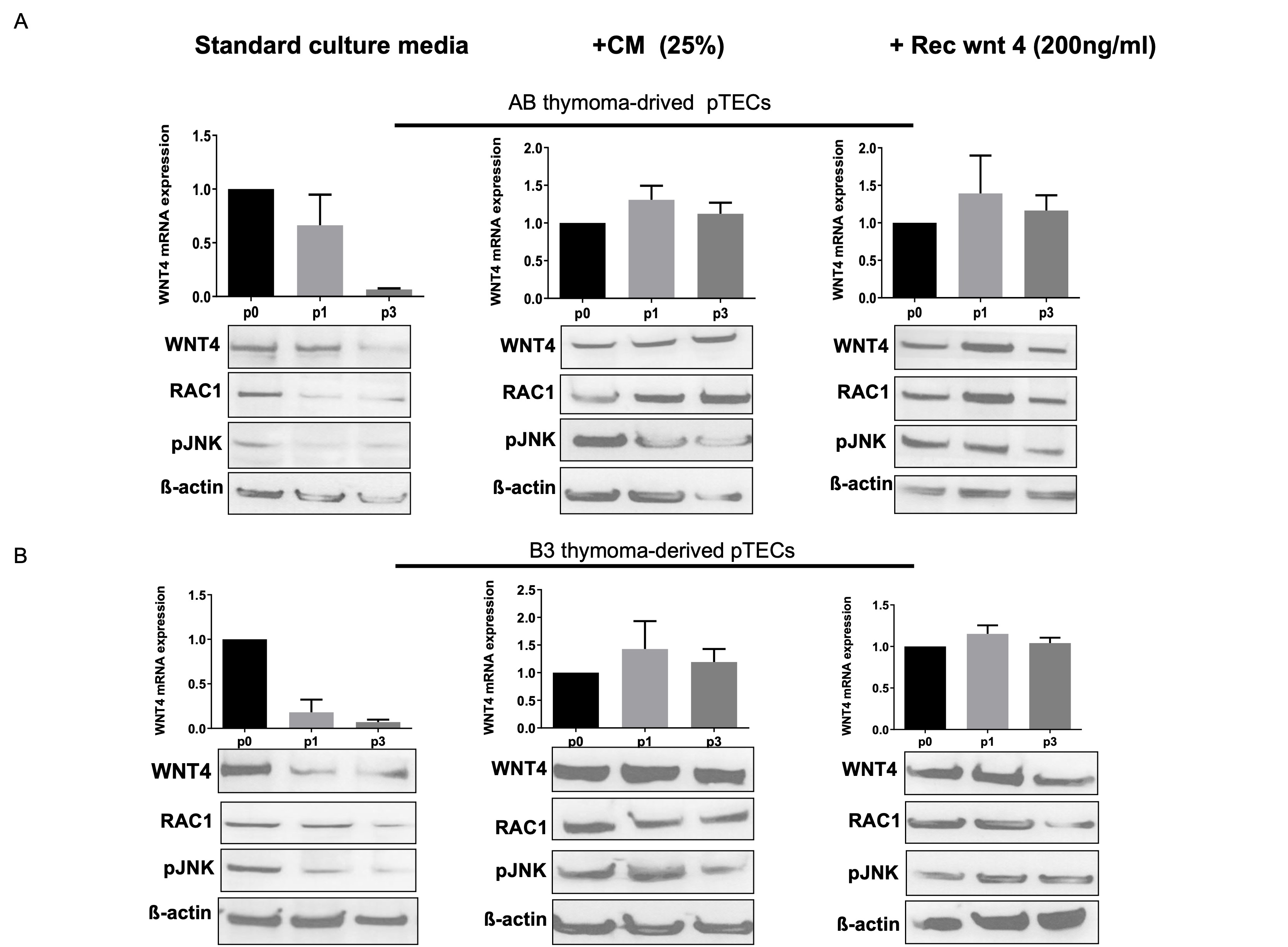


**Supplementary Figure 6.** **Maintenance and increased WNT4 expression in long-term pTECs cultures through stimulation with conditioned medium (CM) or recombinant WNT4.** Slightly increased WNT4 expression in long-term culture of AB thymoma-derived (**A**) and B3 thymoma-derived pTECs (**B**) in standard media enriched with either 25% (v:v) conditioned medium (CM) or 200ng/ml recombinant WNT4 protein (Rec WNT4). RAC1 and its downstream protein phosphor-JNK were maintained in the long-term as well as in the early short-term culture in all neoplastic pTECs. ß-actin is used as a loading control for western blots.

## Supplementary Tables

**Table S1: Real-time PCR primer sequences.**

| Genes | Sequences 5‘-3‘ |
| --- | --- |
| WNT1 | fwd CGGCGTTTATCTTCGCTATC  rev GCCTCGTTGTTGTGAAGGTT |
| WNT2 | fwd GTGGATGCAAAGGAAAGGAA  rev AGCCAGCATGTCCTGAGAGT |
| WNT3a | fwd GGAGAAGCGGAAGGAAAAATG  rev GCACGTCGTAGATGCGAATACA |
| WNT4 | fwd ACCTGGAAGTCATGGACTCG  rev TCAGAGCATCCTGACCACTG |
| WNT5a | fwd GACACCCCATGGCACTTG  rev AGGGCTCCTACGAGAGTG |
| WNT5b | fwd GTGCAGAGACCCGAGATGTT  rev GTCTCTCGGCTGCCTATCTG |
| WNT7a | fwd GCTGCCTGGGCCACCTCTTTCTC  rev GGAGGGTCCTTTTCCTCGGGT |
| WNT7b | fwd TCAACGAGTGCCAGTACCAG  rev CCCTCGGCTTGGTTGTAGTA |
| WNT8b | fwd TCCCAGAAAAACTGAGGAAACTG  rev AACCTCTGCCTCTAGGAACCAA |
| WNT9a | fwd GGGTGTGAAGGTGATCAAGG  rev GCAAGCATCTGAAGCACAAG |
| WNT9b | Fwd TGCACCTGTGATGACTCTCC  Rev CTGATACGCCATGGCACTTA |
| WNT10a | fwd GGCAACCCGTCAGTCTGTCT  rev CATTCCCCACCTCCCATCT |
| WNT10b | fwd GAAAACCTGAAGCGGAAATG  rev GGGTCTCGCTCACAGAAGTC |
| WNT11 | fwd GGCTTGTGCTTTGCCTTCA  rev TTTGATGTCCTGCCCTCCTT |
| WNT13 | Fwd TGCCAAGGAGAAGAGCCTTAAG  rev GTGCGACCACAGCGGTTATT |
| WNT15 | fwd CAGGTGCTGAAACTGCGCTAT  rev GCCCAAGGCCTCATTGGT |
| FZD1 | fwd CAGCACTGACCAATGCCAAT  rev CACCTTGTGAGCCGACCAA |
| FZD2 | fwd TTTCTGGGCGAGCGTGAT  rev AAACGCGTCTCCTCCTGTGA |
| FZD3 | fwd GCTCGGTCATCAAGCAACAG  rev ACGGTGTAGAGCACGGTCAAC |
| FZD4 | fwd GGCGGCATGTGTCTTTCAGT  rev GAATTTGCTGCAGTTCAGACTCT |
| FZD5 | fwd CGCGAGCACAACCACATC  rev AGAAGTAGACCAGGAGGAAGACG |
| FZD6 | fwd CTGGGTTGGAAGCAAAAAGA  rev CCATGGATTTGGAAATGACC |
| FZD7 | fwd CAACGGCCTGATGTACTTTAAGG  rev CATGTCCACCAGGTAGGTGAGA |
| FZD8 | fwd TCTTGTCGCTCACATGGTTC  rev GGTGCCGATGAAGAGGTAGA |
| FZD9 | fwd CTTCTCCACCGCCTTCAC  rev GAAACTACTGCCCAGCACC |
| FZD10 | fwd AGCATCCCCAGAAAACTCAC  rev AACACAACCAAGAAAAGCACC |
| KRT19 | Fwd AATCCACCTCCACACTGACC  rev TTTGAGACGGAACAGGCTCT |
| VANGL1 | fwd TTACCTCCGATCCTGTGGAG  rev AACAAAAGGGCACGAAACAC |
| VANGL2 | fwd CTCGGAGAGGAAAACAGCAC  rev CAGCCGCTTAATGTGAGTGA |
| CELSR1 | fwd AGTGTGGGCCCAGTCACTAC  rev CACTGGCCGTTGGTCTTATT |
| CELSR2 | fwd GTGACTCAAACCCGTGTCCT  rev CTCACAGTATGGCCCAAGGT |
| SFRP2 | fwd GCCTCGATGACCTAGACGAG  rev GATGCAAAGGTCGTTGTCCT |
| SFRP5 | fwd TGGAGCCCAGAAAAAGAAGA  rev GCAGGGGTAGGAGAACATGA |
| DKK1 | fwd ATTCCAACGCTATCAAGA  rev CCAAGGTGCTATGATCAT |
| DKK4 | fwd AGCTCTGGTCCTGGACTTCA  rev CAACCCACGACATGTAGCAC |
| TIMP1 | fwd AATTCCGACCTCGTCATCAG  rev TGCAGTTTTCCAGCAATGAG |
| TIMP2 | fwd AAAGCGGTCAGTGAGAAGGA  rev’ CTTCTTTCCTCCAACGTCCA |
| TIMP3 | fwd CTGACAGGTCGCGTCTATGA  rev GGCGTAGTGTTTGGACTGGT |
| TLE2 | fwd CCAGCGAGAAGACGGAAATG  rev CAGACGCTTCACAATCTCCG |
| TLE4 | fwd GTTTCCGAGGTGCTGAGAAG  rev TAATCGGGGCATCTTTCTTG |
| TCF3 | fwd CATCTGCATCCTCCTTCTCC  rev GAGTAGATCGAGGCCAGTGC |
| TCF4 | fwd ATGCTTCCATGTCCAGGTTC  rev CACTCTGGGACGATTCCTGT |
